# Inferior Vena Cava Filters and Complications: A Systematic Review

**DOI:** 10.7759/cureus.40038

**Published:** 2023-06-06

**Authors:** Joe Bajda, Ann N Park, Aishwarya Raj, Rhea Raj, Vasavi Rakesh Gorantla

**Affiliations:** 1 Anatomical Sciences, St. George's University School of Medicine, True Blue, GRD; 2 Medicine, St. George's University School of Medicine, True Blue, GRD; 3 Vascular Surgery, St. George's University School of Medicine, True Blue, GRD

**Keywords:** trauma, ivcf fracture, permanent ivcf, retrievable ivc filters, pulmonary embolism, venous thromboembolism, thrombosis, ivc filter complications, ivcf placement, inferior vena cava filter

## Abstract

Inferior vena cava (IVC) filters have been used since the 1960s to treat patients with acute risk of pulmonary embolism (PE) to prevent migration of thrombus by trapping it within the filter. Traditional usage has been in patients with contraindication to anticoagulation that carry a significant mortality risk. In this systematic review, we sought to evaluate complications associated with placement of inferior vena cava filters based on published data from the past 20 years. A search was performed on October 6th, 2022, in accordance with the Preferred Reporting Items for Systematic Reviews and Meta-Analyses (PRISMA) guidelines for systematic reviews, using three databases (ProQuest, PubMed and ScienceDirect) for articles published between the dates of February 1, 2002 and October 1, 2022. Results were filtered to include full-text, clinical studies, and randomized trials written in English pertaining to keywords “IVC filter AND complications”, “Inferior Vena Cava Filter AND complications”, “IVC filter AND thrombosis” and “Inferior Vena Cava Filter AND thrombosis”. Articles identified by the three databases were pooled and further screened for relevance based on inclusion and exclusion criteria. Initial search results yielded 33,265 hits from all three databases combined. Screening criteria were applied, with 7721 results remaining. After further manual screening, including removal of duplicate hits, a total of 117 articles were selected for review. While there are no consensus guidelines for best practice, there is compelling evidence that IVC filters can provide significant protection against PE with minimal complications if the treatment window is appropriate. Increase in the variety of filter models has led to broader availability, but skepticism remains about their efficacy and safety, with ongoing controversy surrounding appropriate indications. Further research is needed to establish clear guidelines on appropriate indications for IVC placement and to determine time course of complications versus benefits for indwelling filters.

## Introduction and background

Inferior vena cava (IVC) filters are implantable devices placed by vascular surgeons or interventional radiologists into the inferior vena cava to prevent the migration of a thrombus to the pulmonary vasculature [1]. Filters are typically inserted through the femoral, jugular, or antecubital veins, with the infrarenal IVC as the primary target of placement. Placement is performed with imaging assistance to ensure proper guidance. There are two general types of filters - permanent and retrievable. The scope of indications for IVC filter placement, however, remains controversial because of lack of definitive evidence supporting mortality benefit beyond the traditional indication of a patient with significant pulmonary embolism (PE) risk who is contraindicated for anticoagulation. IVC filter use has also been expanded due to the availability of retrievable filters without any definitive changes in guidelines for their use [2]. In accordance with the Society of Interventional Radiology (SIR), indications for placement include: (1) for patients with documented venous thromboembolism (VTE) and classic indications including: contraindications to anticoagulation, complication of anticoagulation requiring discontinuation, failure of anticoagulation, and worsening of deep vein thrombosis (DVT) during anticoagulation treatment; (2) for patients with documented VTE and expanded indications including: iliocaval or large free-floating proximal DVT, inability to maintain sufficient anticoagulation, massive PE with remaining DVT and recurrent risk for subsequent PE, recurrent PE with IVC filter placement, VTE with limited cardiopulmonary reserve, patients at high risk of complications from anticoagulation, and chronic VTE treat with thromboendarterectomy; and (3) for primary prophylaxis in patients without VTE. The most common indication is for patients with a history of VTE that have a contraindication to anticoagulants [2-5]. Specific indications for IVC filters are variable depending on organizations and their guidelines. According to the European Society of Cardiology, IVC filters are not recommended for prophylactic placement, for free-floating thrombus, or prior to systemic thrombolysis, surgical embolectomy, or pulmonary thromboendarterectomy. The only agreed-upon indication appears to be patients who have a past history of VTE, are at high risk of PE, and who have a contraindication to anticoagulation treatments [6]. 

Permanent IVC filters, meant to be left in the patient indefinitely, were first established for use in patients with VTE in 1967 with the introduction of the Mobin-Uddin group filter. Permanent filters offer no option of removal, so are only indicated for patients with long-term contraindication to anticoagulation therapy. Retrievable (temporary) filters, invented in the 1990s, could be implanted during the critical period with an option of removal prior to discharge [7,8]. The possibility of removal has expanded indications for filter placement, with device retrieval only occurring sporadically [2,7]. Currently there are several filter options available which include permanent, retrievable and convertible filters [7]. Bio-convertible filters, such as the Sentry (Boston Scientific, Marlborough, MA, USA) and VenaTech (Braun, Bethlehem, PA, USA) models, are reported to provide temporary (~60 days) protection against PE before retraction of the filter arms and stent-like incorporation into its surrounding vasculature, thus eliminating the need for an additional removal procedure while avoiding potential complications of indwelling filters like filter tilt, fracture, or IVC penetration [9-11]. In 2012, the new Healthcare Common Procedure Coding System introduced new codes for claims specific to IVC filter placement and removal, which allowed better tracking of these procedures. Ahmed and colleagues performed a study on Medicare claims based on these new codes in the years 2012-2016 and found that filter placements declined from 61,889 cases in 2012 to 38,095 in 2016, while retrieval rates increased from 4327 in 2012 to 8405 in 2016 [12]. 

Given the lack of consensus around best practices and efficacy of IVC filters, this systematic review was written to examine data regarding mortality and complications related to IVC filters, and the indications for which they demonstrate the most benefit. Many patients in whom IVC filters are placed are considered high risk, and this complicates determining whether filter placement improves patient outcomes. This paper includes data pertaining to filter complications ranging from insertion site hematoma, to filter migration and fracture. Complications and safety of IVC filter therapy are reviewed using endpoints of mortality risk and recurrence of thrombotic complications, primarily PE. Data regarding various filter models are included for comparison and context. Finally, information regarding common clinical settings, such as patients undergoing cancer treatment, bariatric surgery, and orthopedic surgery, are included in the scope of this review. The aim of this paper is to gain deeper insight into the complications and safety of IVC filters in major use cases. Despite a dearth of randomized control trials related to the effectiveness of IVC filters, our goal is to parse existing research to clarify settings in which IVC filters reduce complication rates and remain safe.

## Review

Methods 

This systematic review follows the Preferred Reporting Items for Systematic Reviews and Meta-analyses (PRISMA) guidelines [13]. Database searches were performed on October 6, 2022 (PubMed, ProQuest, and ScienceDirect) for articles published between February 1, 2002 and October 1, 2022. Queries were made using the keywords, “IVC filter AND complications”, “Inferior Vena Cava Filter AND complications”, “IVC filter AND thrombosis”, and “Inferior Vena Cava Filter AND thrombosis”. The original searches before adding exclusion criteria yielded 25,972, 7000, and 291 results from ScienceDirect, PubMed, and ProQuest, respectively. This gave a total of 33,265 results. Articles not written in English, without full text, and published before 2002 were excluded, leaving 7721 remaining articles. Manual screening and removal of duplicates were performed using the exclusion criteria defined below, based on article title, abstract, study type, relevance, and full-text availability. Three co-authors independently screened results ultimately leading to a total of 117 articles included in our review. 

Inclusion Criteria 

Inclusion criteria for articles were full-text, peer-reviewed, written in English, published or in press between February 1, 2002 and October 1, 2022, clinical trials, meta-analyses, original studies, observational studies, retrospective studies, and articles relevant to our topic of interest (IVC filter and related complications). 

Exclusion Criteria 

**Figure 1:**
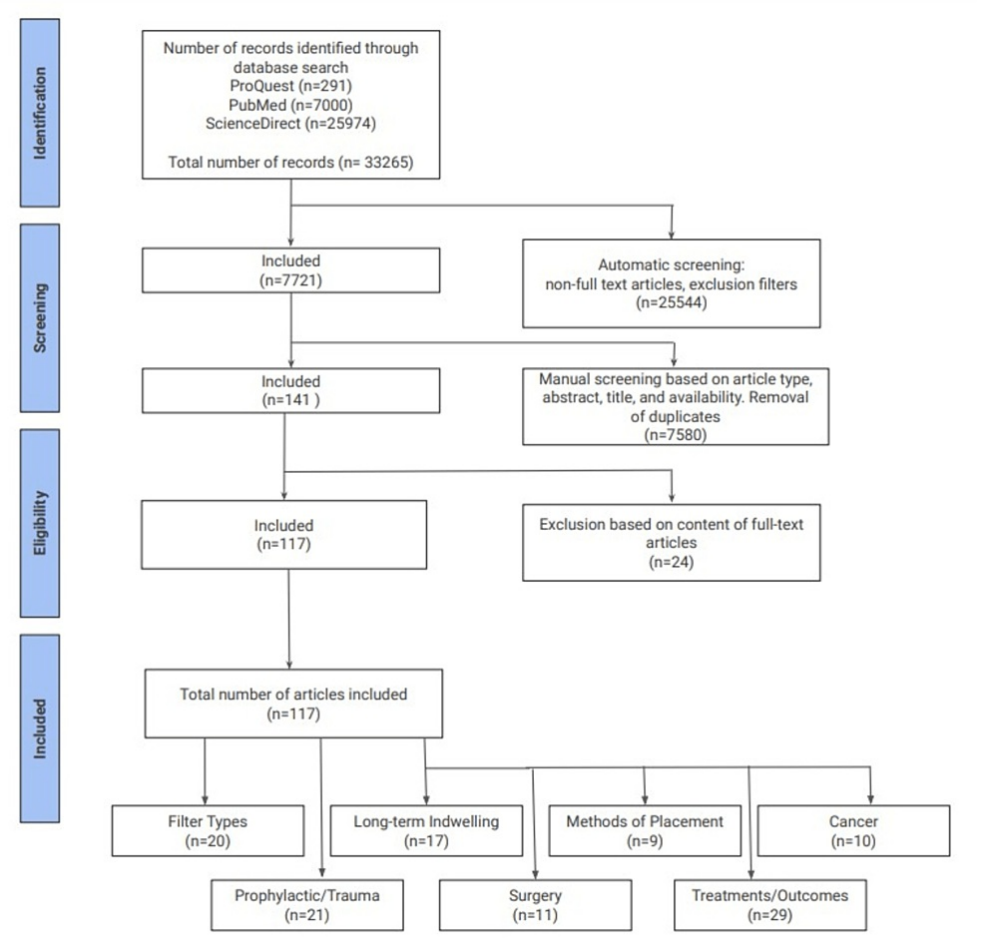
Preferred Reporting Items for Systematic Reviews and Meta-Analyses (PRISMA) flow chart. Summary of exclusion criteria used to narrow down search results, and categorization of articles included in this systematic review.

Exclusion criteria included systematic reviews, review articles, case studies, case reports, studies on non-human subjects, articles related to filter retrieval complications, and articles published before 2002. Non-full-text articles and duplicate articles were excluded. The search process, considering inclusion and exclusion criteria, is illustrated in the PRISMA flow diagram below (Figure 1). 

Risk of bias in selected studies 

Selected studies were reviewed by three co-authors to rate the risk of bias using the Grading of Recommendations Assessment Development, and Evaluation (GRADE) system. GRADE evaluates studies by determining the risk of bias, indirectness, imprecision, and publication bias. Two of the co-authors reviewed each study individually using the GRADE criteria, while the third reviewer assessed and mediated an outcome if there were any inconsistencies. 

Results of literature searches 

A review of literature was performed inclusive of three medical databases, ScienceDirect, PubMed, and ProQuest. Our initial search resulted in 33,265 articles: 25,974 from ScienceDirect, 7000 from PubMed, and 291 from ProQuest. Automatic inclusion of articles using database filters including: meta-analysis, clinical trials, randomized control trials, full-text articles, and those published between February 1, 2002 and October 1, 2022 removed 25,544 hits from the results pool. After further manual screening based on article type, title, abstract content, availability, and removal of duplicates, 7580 additional articles were excluded. Three co-authors reviewed the full text of the remaining pool of 141 articles and excluded 24 additional articles based on content relevance. Ultimately, 117 articles were deemed relevant to this systematic review, and categorized into discussions of filter types, long-term indwelling and complications, IVC filters in cancer patients, IVC filters in patients undergoing surgery, or IVC filters in the context of prophylaxis and/or trauma. Of these included articles, we reviewed five meta-analyses, 10 prospective studies, four clinical trials, 92 retrospective studies, four randomized control trials, one observational study, and one population-based study. 

Results and discussion 

Virchow’s Triad can be used to explain thrombogenesis and propagation. The triad consists of hypercoagulability, stasis of flow, and endothelial damage. Hypercoagulability, also known as thrombophilia, relates to the constitution of blood that increases risk of thrombus formation. Various clinical and disease statuses are associated with hypercoagulability such as pregnancy, use of oral contraceptives, protein C deficiency, protein S deficiency, homocystinuria, and others. Stasis refers to alterations that typically decrease velocity of blood flow. It is believed that slowed blood flow reduces exposure to cell proteins that trigger a natural anticoagulation pathway, resulting in thrombus formation. Stasis can be seen in several situations such as atrial fibrillation, prolonged immobility, extensive surgery, or long travel. Injury to the vascular wall can cause alterations in normal blood flow. Flow disturbances create disordered currents that increase friction of flow through a vessel. Endothelial damage results from a variety of factors: smoking, atherosclerotic disease, chronic hypertension, inflammation, medical devices, etc. [14]. IVC thrombosis has commonly been associated with IVC filters. Following Virchow’s Triad, IVC filters can affect both stasis and the endothelium, resulting in a possible increased risk of thrombus formation. 

Methods of Filter Placement 

Prior to 1989, all IVC filters were placed by vascular surgeons. Complications can arise during the placement of the filter itself. Bleeding and acute thrombosis at the surgical site are most common. Immediately after placement, filter tilt, which is defined as angulation greater than 15 degrees from the longitudinal axis of the IVC, and filter migration, change in position more than 2 cm, can also occur [15]. Human error can also be a cause of procedure complications such as misplacement or disorientation. The quantity of IVC filters utilized increased dramatically when the percutaneous delivery method was introduced, allowing interventional radiologists to predominate as the primary clinicians involved in filter placement. Lin et al. retrospectively analyzed 592 patients who underwent filter placement between March 1987 and December 2000. Complications such as insertion site hematoma or DVT, filter migration, and IVC thrombosis were compared between those who had operative IVC filter placement by vascular surgeons and percutaneous delivery by interventional radiologists. Complication rates between the groups were not statistically different (P=0.48). No mortality was directly related to filter placement. Chronological analysis revealed that while radiologists rocketed to become the primary clinicians in filter placement after 1989, introduction of an endovascular program for vascular surgeons in 1994 resulted in a small resurgence of filter placement by surgeons with continuous steady increases. Based on the similar complication rates, Lin et al. suggest that a multidisciplinary team of radiologists and surgeons would provide optimal patient care in IVC filter placement [16]. 

Typically, IVC filters are placed guided by fluoroscopy in dedicated angiographic suites. Ganguli et al. sought to compare the safety and associated complications of placement via ultrasound at bedside versus traditional fluoroscopy. Between 2009 and 2011, 117 patients received IVC filters at bedside in the ICU and 571 patients underwent fluoroscopy-guided placement. Complications related to placement occurred in 4.3% of patients who received bedside filters. These complications included four cases of malpositioning and one severe tilt. Fluoroscopy generated one malposition and one severe tilt resulting in a complication rate of 0.6% (P=0.01). Complications related to indwelling time occurred similarly between both groups. Median indwelling time to complication was 74 days for the ultrasound cohort and 127 days for the fluoroscopy group. In the ultrasound group, DVTs occurred in 13.7%, PE in 5.1%, and filter thrombosis in 3.4% of the cohort. Comparatively, the fluoroscopy group resulted with DVTs in 13.3%, PE in 4.0%, and filter thrombosis in 3.9% (P=0.92, P=0.61, P=0.82, respectively). Ultimately, this study concluded that placement at bedside guided by ultrasound is a safe procedure but is associated with more complications than fluoroscopy [17]. 

There is difficulty in placement of filters in patients who have suffered head trauma and require intracranial pressure monitors as these patients are unable to lay supine for traditional fluoroscopy or beside ultrasound. Searching for a safe alternative, Joseph and Lopera performed a retrospective review in 2021 analyzing digital radiograph (DR) guided filter placements at ICU bedside in patients with increased intracranial pressure (ICP). A total of nine filters were placed guided by DR (eight with indication for prophylaxis, one for acute DVT). Filters placed included four Denali (Becton Dickinson, Tempe, AZ, USA), three Option Elite (Argon Medical Devices, Plano, TX, USA), and two Celect (Cook Medical, Bloomington, IN, USA). Two deaths occurred that were not related to the procedure. All nine filters were placed successfully at the level of the lowest renal vein. Average pre-procedure ICP was 16 mmHg, procedural was 13 mmHg, and post-procedure ICP was 16 mmHg. Thus, there was no significant difference in pre and post-procedural ICP (P=0.77). Filter tilt was reported as a complication in one of the Option Elite filters. Joseph and Lopera found that DR is a safe alternative method of IVC filter placement, primarily indicated in patients with increased intracranial pressure who are unable to lay supine [18]. Walker et al. examined a cohort of 129 patients, 48 who received filters guided by DR at bedside and 81 who underwent traditional fluoroscopy. Filter positioning and tilt were evaluated with post-procedural cavograms and Computerized Tomographs (CTs). Both groups experienced 100% technical success in placement. Bedside placement did have a significantly longer procedure duration compared to fluoroscopy: 14.5+/-10.2 versus 6.7+/-6.0 min (P<0.0001). However, DR at bedside was associated with significantly reduced radiation exposure: 25 mGy (15-35) vs 256.94 mGy +/- 158.6. Common complications did occur in both groups but were not significantly different: malpositioning (P=0.31), degree of filter tilt (P=0.33), and other complications (P=0.65). The authors concluded that there was no significant difference in outcomes when comparing bedside DR to traditional fluoroscopy placement [19]. 

A common complication that tends to arise with IVC filters is mechanical tilt of the filter during or after insertion. Xiao et al. sought to determine if utilizing the introducer curving technique could aid in reducing filter tilt during transfemoral insertion of Gunther Tulip filters (Cook Medical). One hundred eight patients receiving IVC filters were randomly divided into Group C and Group T, with Group T adopting the introducer curving technique. The post-implantation filter tilting angle and adherence of retrieval hook to the vascular wall was measured and compared between groups. Average post-implantation filter tilting angle in Group C was found to be 7.1±4.52 degrees, and in Group T was 4.4±3.20 degrees. Thus, the study found a statistically significant difference in average post-implantation filter tilting angle between groups (t=3.573, P=0.001). There was also a statistically significant difference in post-implantation filter tilting angle between right and left approaches (5.1±3.82 vs. 7.1±4.59, t=2.301, P=0.023). Additionally, Group T displayed a significantly lower rate of severe tilt (post-implantation filter tilting angle ≥ 10o) than Group C (9.3% vs. 24.1%, χ2=4.267, P=0.039). Retrieval hook adherence was also found to be statistically significant between Group T and Group C (2.9% vs. 24.2%, χ2=5.030, P=0.025, respectively). Ultimately, Xiao et al. found that the introducer curved technique of insertion was effective in reducing filter tilt during transfemoral insertion of the Gunther Tulip filter [20]. 

Choi et al. analyzed complications associated with Denali filter placement at different venous access sites. Three access points were compared: right femoral, left femoral, and right jugular. Variables measured included IVC diameter, degree of filter tilt, filter tip abutment/limb penetration, fluoroscopy time, and retrieval. The cohort consisted of 78 patients who had previously received Denali filters in successful placement. Seventy-one patients had both pre-procedural and pre-retrieval CTs for comparison. Thirty-five patients received filters via right femoral placement, 22 at the left femoral vein, and 14 at right jugular. Sixty-eight cases had attempted retrieval, all of which were successful. There was no significant difference in abutment or filter tilt among the three access sites. Abutment occurred in eight of the 71 patients with comparative CTs. Filter limb penetration occurred in two of the 71 patients. Other than one filter fracture that occurred during advanced retrieval, no other complications were observed. One variable that did result in a significant difference was length of fluoroscopy. Placement through the right jugular vein had an average fluoroscopy time of 117+/-37 s compared to right and left femoral placement (64+/-21 s and 67+/-15 s, respectively). All three access points were found to be similar in terms of associated complications, with a jugular approach being associated with longer fluoroscopy time [21]. Impact of access point was also studied by Grullon et al. who compared transjugular insertion with transfemoral. This study found increased risk of filter angulation (0.9% vs 0.34%; odds ratio [OR] 1.46 confidence interval [CI] 1.02-15 2.11; P=0.04) and rate of access site complications (0.25% vs 0.07%; OR 2.068; CI 1.01-4.23; P=0.048) in the transfemoral insertion group, with no significant difference in difficulty of retrieval between the groups [22]. It is important to note that although angulation rates and access site complications were higher in the transfemoral group, both insertion sites exhibited a low frequency of these complications. Both methods seem to be viable with specific patient anatomy and presentation playing a role in clinical decision making. Furthermore, trans popliteal insertion of IVC filters was found to be safe and efficient in a small retrospective study (n=21) performed by Kim et al. [23]. 

The safety of placement and retrieval of suprarenal IVC filters was analyzed by Baheti et al. In this retrospective study, 51 filters (40 Gunther Tulip, 10 Denali, and one Celect) were placed in the suprarenal position. Indications for this position included IVC thrombus, anatomical variants, and external IVC compression. Twenty-seven retrievals were attempted, all of which were successful; however, one retrieval was complicated with fracture struts. The median indwelling time was 87 days. No filter tilt or fracture was observed while the filter remained in place. There was also no significant change in renal function [24]. This study confirmed that if indicated, suprarenal positioning of IVC filters can be done safely and with low complications. 

Outcomes: Filter-Related Complications 

IVC filters are placed in order to prevent emboli from traveling to the pulmonary vascular bed. Complications with the filter may arise during placement, post-procedure, and during retrieval. Filter fracture and perforation are among the most common causes of failure, each with a positive correlation to longer indwelling time [25,26]. Indications for IVC filters vary widely within and across institutions. However, the types of filter-associated complications that arise are consistently seen across most studies. 

Filter-related IVC perforation is defined by penetration of the IVC filter >3 mm into the wall of the vena cava, which accounts for about 20% of IVC filter complications per the MAUDE database. Retrievable filters have a higher rate of perforation, with most cases largely asymptomatic [27]. Asymptomatic perforations are often found incidentally on abdominal CT [28]. Similarly, there have been case reports in which patients were asymptomatic and perforation was noted incidentally during other intra-abdominal surgeries [29]. Perforations may present symptomatically within days to weeks. The primary complaint related to IVC perforations appears to be vague abdominal pain. Symptomatic cases accounted for one in 10 perforations and mostly required endovascular retrieval [30]. 

Fracture of filter limbs is directly related to the indwelling time of the filter. In very rare cases, the filter fragments can travel through the IVC and embolize in the heart or lungs. Fragments that travel to the right heart can present similar to pulmonary embolisms. Symptoms include shortness of breath, chest pain, and syncope. A case study by Thakur et al. from 2015 reported a linear structure moving with cardiac motion in the proximal right ventricle in a patient with chest pain. Fluoroscopy was used to visualize the IVC filter which showed normal position but visible fractures of at least two of the filter legs. The patient was made aware of the fractures and educated on possible risks regarding removal of the filter. The patient elected not to have the surgery performed and the fragment was left in situ [31]. The relative risk of fracture among different filters is currently a subject of study. Further investigation into fracture-prone filters will be able to assist clinicians in their medical decision-making. 

Thrombotic complications are not uncommon following IVC filter placement. Duffet’s et al. study of 338 patients at two Ottawan tertiary care hospitals reported that 68 patients (20% total) had one or more filter-related complication, with 38 patients (11%, 95% CI 8.2-15.0) of total patients having one or more thrombotic complications that included IVC filter thrombosis (7%, 95% CI 5.0-10.6), recurrent DVT (5%, 95% CI 3.1-7.8) and recurrent PE (3%, 95% CI 1.7-5.6). Despite the fact that most filters (91%) were placed appropriately for contraindication or discontinuation of anticoagulation, the high rate of thrombotic complications underscores the need for further research regarding the benefits of IVC filters [4]. 

Various single-center studies in different countries report rates of 10-20.6% of total filter complications with a large proportion attributed to thrombotic complications. 

Shabib et al.’s report on a single Saudi Arabian facility over 11 years similarly found a complication rate of 20.6% in 382 filter insertions. Of these patients, recurrent DVT occurred at the highest rate (39%) followed by IVC thrombosis (32%), new/recurrent PE (18%) and other thrombosis (11%). Mechanical complications occurred in only 1.8% (seven patients), which consisted of filter tilting in six patients and IVC occlusion in one patient [32]. Nazzal et al.’s single institution study in Ohio consisted of 400 patients over a four-year period and found a 12.6% rate of complications, mostly due to thrombotic events; IVC thrombosis (4.75%), ipsilateral DVT (3.8%), PE (1.5%), filter migration/tile (1.5%) and hematoma at filter insertion site (1%) [33]. Wassef et al.’s study found 464 patients with IVC filters inserted over four years at a facility in Alberta, Canada. Overall IVC filter-associated complication rate was 22.2% (103). The most common complication was filter thrombosis (12.5%) and filter tilt (9.5%) [34]. Weinberg et al.’s study of a single Oklahoma (USA) center over three years reported 758 IVC filter placements. Insertion-related complications occurred in 4.1% of patients, accounted for by malpositioning and filter angulation. Filter-related complications within the first 32 days occurred in 19% of patients; 10.5% DVT, 4.2% PE, 3.8% IVC thrombosis [35]. A single-center study in Kyoto, Japan of 257 patients over eight years had a 10% rate of IVC filter-related complications that included 2.3% thrombus occlusion and single instances of infection and filter malpositioning [36]. 

In a study done by King et al. of 5780 IVC filter insertions across 62 US and Canadian facilities, they found a relatively low rate of IVC filter thrombosis (78 patients, or 1.3%) with two-year follow-up. However, based on their analysis of factors associated with filter thrombosis, they found an association with lack of antiplatelet therapy (hazard ratio 4.8, 95% CI 1.9-12.5, P=0.001) leading to their primary conclusion that antiplatelet therapy should be considered as a preventative measure against IVC filter thrombosis formation [37]. 

Hammond’s report on 507 IVC filter placements in three UK centers over 12 years reports low complication rate associated with filter insertion (1.7%) despite an increasing trend of filter placement that mirrors US trends. There were two major complications (0.4%). One patient died in post-procedural recovery thought to be caused by PE. The second was IVC perforation within several hours of filter insertion, which required an emergency laparotomy. The remainder consisted of inadvertent placement, technical difficulty and wound oozing. Thirteen (2.6%) filtration-related complications occurred, including IVC occlusion (six patients, 1.2%), recurrent PE (four patients, 0.8%), infection (two patients, 0.4%), and filter migration (one patient, 0.2%). 24-hour and 30-day mortalities were 1 and 8%, and non-filter related [38]. 

Jung-Kyu et al. performed a retroactive observational study of 45 patient records from a single Korean institution over nine years for IVC filter-related complications based on appropriate follow-up CT imaging in patients with baseline presence of DVT and/or PE. The most common complication was IVC penetration (86.7%) and filter tip embedding suggestive of lateral tilting (51.1%). Filter thrombosis was suspected in 20% of patients. They found a 15-fold increased risk of significant IVC filter penetration if filters were left indwelling beyond 20 days (95% CI 3.6-68.7). However, there was no symptomatic complication in any of the reviewed charts [39]. 

Temporary IVC filters are the newest addition to the IVC filter market and as such, have the least amount of data regarding their utility or complications. One report came from Tokyo University Hospital (Miyahara) where two different temporary filters (Neuhaus Protect [Toray Medical, Tokyo, Japan] and Antheor [Boston Scientific]) were implanted in patients for various indications; 9.1% were contraindicated for anticoagulation, 12.1% for thrombolytic therapy, 84.8% perioperative prophylaxis, 3% DVT in pregnancy, and 15.2% prophylaxis without evidence of DVT. Though there was no incidence of PE-associated mortality, major complications arose in 27.3% of patients. These included 12.1% filter dislocation, fractured catheter in 9.1%, and catheter-related infection in one patient [40]. 

Clements et al.’s study investigated the interesting question of whether IVC filters themselves are thrombogenic in nature and if this necessitates concomitant anticoagulation. The single institution study included 124 patients who received prophylactic IVC filters in the setting of major trauma. No patient resulted with IVC thrombosis during the time of filter indwell. Their findings suggest that IVC filter implantation alone does not indicate need for anticoagulation. Authors corroborate current practices, in which thrombotic events indicate need for therapeutic anticoagulation [41]. 

Ramakrishnan et al. performed a retrospective study of 14,784 patients under the Vascular Quality Initiative Registry. In this study, IVC filter complications occurred at a rate of 1.8% (immediate) vs 3.1% (delayed), though neither immediate nor delayed IVC filter-related complications were associated with increased mortality rate [42]. Interesting to this article is that the authors suggest actionable measures that could reduce the incidence of both immediate and delayed complications. Immediate complications (which include filter placement, misplacement, or insertion site-associated injury) can be avoided by early utilization of advanced imaging of the renal vein to ensure proper placement and deployment. Delayed complications (which include filter tilting/fracture/migration, thrombus formation/embolization, or vessel perforation) can be reduced given diligent follow-up and timely removal, implying proper follow-up protocols must be established at an institutional level. It is reasonable to argue that if these changes can demonstrably reduce complications, they should be implemented because patients receiving IVC filters are often already complicated or critical at the time of filter placement. 

There are a set of complications that can occur during filter retrieval which are positively correlated with indwelling time. Filter fracture and IVC injury upon removal are possible outcomes [7,25,39,43,44]. These complications are outside the scope of our investigation and have been excluded in this article. 

Outcomes: Mortality 

Without randomized controlled trials to guide clinical decisions, a number of studies have relied on data from the National (Nationwide) Inpatient Sample (NIS) to evaluate IVC filter-related outcomes. The NIS is part of the Healthcare Cost & Utilization Project and contains information on over seven million hospital stays in the U.S. annually. The NIS is the largest publicly available healthcare database for informed decision-making on local, regional and national levels. NIS data is currently available for years 1988-2019. Since 2016, NIS data can be identified with ICD-10-CM/PCS diagnosis and procedural codes. 

Dr. Paul Stein and colleagues have published several studies suggesting that certain populations of patients may derive mortality benefit from IVC filter implantation. 

When analyzed by age group, Stein et al. (2016) found that IVC filters placed for primary diagnosis of PE without thrombolytic therapy modestly decreases mortality for patients of age >80 years old, even when accounting for comorbidities according to the Charlson Comorbidity Index [45]. Stein et al. (2017) did a follow-up study with data from the Premier Healthcare Database on patients >60 years hospitalized with PE and solid tumor malignancies. Among certain types of solid tumors, in-hospital all-cause mortality was lower in patients who had IVC filters (7.4%) vs. patients without (11.2%) (P<0.0001, relative risk 0.67). They also found a slightly lower three-month all-cause mortality with filters (15.1%) than without (17.4%) (P<0.0001, relative risk 0.86) [46]. 

Stein et al. (2019) investigated if IVC filter-related outcomes can be better seen when patients with PE are subdivided into stable or unstable categories, with unstable defined as in shock, or dependent on a ventilator. After stratification, unstable patients with an IVC filter have a greater reduction in mortality than those without (28.8% vs 46.3%, P<0.0001). Stable patients on the other hand had a lower, albeit not clinically meaningful, reduction in mortality rate than those without (5.8% vs 6.5%, P<0.0001) [47]. When controlling for immortal time bias, Stein (2018) found that regardless of thrombolytic therapy, unstable patients who received an IVC filter had a lower in-hospital all-cause mortality than those without (19.4% vs 40.8%, P<0.0001). Of note, the study mentioned that reduced mortality was associated with filters placed within one or two days of admission [48]. In both stable and unstable patients having undergone pulmonary embolectomy, Stein et al. (2020) found a reduction in mortality with an IVC filter; 4.1% vs. 27% (stable), 18% vs 50% (unstable), P<0.0001 for both sets. Here, mortality was improved only when filter insertion occurred within the first four to five days of admission [49]. 

Gul et al. studied IVC filter placement in PE complicated by pathologies like heart or respiratory failure, shock, and thrombolytic intervention. Patients with and without IVC filter placement with complicated PE were 1:1 propensity score matched for demographics, DVT, Elixhauser Comorbidity Index and other PE comorbidities. Their study found that IVC filter placement reduced mortality rates overall and in each subgroup, corroborating Stein et al.’s findings that IVC filters may provide greater benefit in gravely ill patients [50]. 

In recurrent PE, Stein et al. (2019) found that IVC filter insertion was associated with improved mortality in patients within three months of an index PE (3.0% with vs 39.3% without, P<0.0001). Specifically in the setting of recurrent PE without thrombolytic therapy or pulmonary embolectomy, stable patients may derive greater mortality benefit with an IVC filter than in other situations (2.6% with, 42.6% without, P<0.0001) [51]. 

Liang et al. looked specifically at short-term in-hospital mortality in patients with acute PE but found that the presence of IVC filter did not decrease mortality hazard for patients with acute PE than those without IVF filter (hazard ratio 0.93, 95% CI 0.89-1.01). Similar results were obtained for filter presence in high-risk patients with or without thrombolytic therapy (hazard ratio 0.85, 95% CI 0.6-1.21). Studies were done using propensity-weighted extended Cox analysis. Like Stein’s 2018 and 2019 studies, Liang et al. accounted for immortal time bias and used similar NIS data, albeit with a slightly shorter time frame (Stein used years 2009-2014 whereas Liang’s data was from 2009-2012). However, their conclusions differ as to whether IVC filters improve in-hospital mortality outcomes [52]. 

In a single hospital study of 248 patients, Jha et al. found that although an IVC filter was more likely placed when a patient had right heart strain or DVT (both P<0.001), there was no statistically significant difference between in-hospital mortality of patients with or without an IVC filter and acute PE (P=0.37) [53]. 

A systematic review and meta-analysis by Liu et al. elaborates on the limited potential benefit of IVC filters in patients with PE. The paper analyzed six studies from the USA, France and Australia assessing the use of IVC filters in 1274 adult patients with DVT and/or PE. Filter presence made no statistical difference in PE-related mortality (P=0.81) or overall mortality (P=0.13) within three months of filter placement or throughout the whole follow-up period up to eight years (PE-related mortality P=0.81, overall mortality P=0.61). They did, however, find that patients with IVC filters had an overall lower rate of PE occurrence than those without (3.2% vs 7.79%, 95% CI 0.25-0.71, P=0.001). Filter presence also reduced PE occurrence in patients at high risk of PE (P=0.01) and with absolute contraindication to coagulation (P=0.04). The placement of IVC filters had no significant effect on new incidence of DVT (P=0.58) [54]. While this paper and others question the survival benefit of IVC filters, Liu et al.'s findings suggest that filters may provide some protection against occurrence of PE in VTE patients. 

Outcomes: Adjunctive Therapy 

Though not an officially recommended usage, IVC filters are often used as adjunctive therapies to traditional anticoagulant or thrombolytic therapies which therefore warrants evaluation. 

Isogai et al. investigated in-hospital mortality outcomes of 13,125 patients across 1015 acute care facilities in Japan who were hospitalized for PE, and upon admission, received antithrombotic or anticoagulant therapy with or without an adjunctive IVC filter. They found that patients who had a filter placed had a significantly lower in-hospital mortality rate (3948 patients, 2.6%) than those who did not (9177 patients, 4.7%, P<0.001, risk ratio 0.55, 95% CI 0.43-0.71). This finding was consistent after controlling for age, sex, pre-existing conditions, severity of disease, and therapeutic interventional procedures [55]. 

For patients with recurrent VTE within three months of starting anticoagulation therapy, Mellado et al.’s study suggests a more nuanced risk vs benefit of implementing IVC filters into treatment. This cohort study was assembled from the RIETE registry (Registro Informatizado de la Enfermedad Tromboemólica) and propensity score-matched groups were compared for survival benefit. They found a marked decrease in all-cause death for patients whose VTE recurrence presented as PE (2.1% vs 25.3%, P=0.02). However, placement of IVC filters was not significant for reduction of mortality in patients whose VTE recurrence presented as DVT, nor for PE-related mortality [56]. 

Another cohort study by Muriel et al. identified a patient population (n=344) from RIETE, looking for IVC filter-related survival benefit in patients with acute symptomatic VTE and significant bleeding risk. Specifically, they analyzed clinical outcomes of mortality (all-cause and PE related) as well as recurrent VTE within 30 days of treatment between propensity-matched groups. After comparison of a 1:1 match of patients treated with and without IVC filters they found no significant difference in all-cause mortality, a slight decrease in risk for PE-related mortality with a filter (1.7% vs 4.9%, P=0.03), and increased VTE recurrence with presence of filter (6.1% vs 0.6%, P<0.001) [57]. 

Outcomes: Prophylaxis Before Catheter-Directed Thrombolysis (CDT) and Percutaneous Endovenous Intervention (PEVI)

IVC filters have been temporarily implanted in patients prior to catheter-directed thrombolysis procedures but several studies suggest that without IVC filters, there is a high risk for iatrogenic PE. 

Kolbel et al. aimed to measure the frequency of filter embolization in 40 patients who underwent catheter-directed thrombolysis (CDT) for proximal DVT. After evaluating sequential phlebograms, visible emboli were found in 18 (45%) of patients ranging from <1 cm to >1 cm in size. Filters were removed after CDT and no patient developed symptomatic PE or significant filter-associated complications. Interestingly, patients with an underlying hypercoagulable disorder had fewer cases of IVC filter embolization than patients without (31% with, 69% without, 95% CI 0.02-0.56, P=0.006). Further analysis showed no significant differences in patient backgrounds or procedural factors [58]. Jiang et al. did a similar study with a slightly larger population of 189 patients undergoing CDT for acute proximal DVT, but in this group only eight of 189 patients (4.2%) were found to have IVC filter thrombus. No patient developed symptomatic PE following CDT and filter retrieval, procedural or thrombotic complication [59]. 

Akhtar looked at the adjunctive use of IVC filters in patients undergoing CDT for proximal lower extremity or caval DVT (NIS database, Jan 2005 - Dec 2013) and found no improvement in mortality on the basis of IVC filter placement (0.7% vs 1.0%, P=0.2). Moreover, Akhtar suggests that IVC filters may actually have increased burden on patients and hospitals given that filter placement was associated with higher rates of hematoma, in-hospital costs, and durations of admission [60]. 

Sharifi et al.’s study suggests that unlike CDT, prophylactic IVC filter usage may provide therapeutic benefit in percutaneous endovenous intervention (PEVI) for therapeutic removal of acute proximal DVT. In this study, they found that IVC filters reduced the incidence of iatrogenic PE (P=0.048). This was a single study of 141 patients, and warrants further investigating the role of IVC filters in conjunction to the procedure [61]. 

Comparison of Filter Types 

Many filter types have been studied, varying from permanent to replaceable and bioconvertible. Peer-reviewed articles from our searches suggest that IVC filter placement, particularly retrievable filters, can be performed safely, with better outcomes in terms of mortality and recurrence of PE, and with minimal complications. TrapEase (Cordis, Miami Lakes, FL, USA), Denali, Recovery (Becton Dickinson), Sentry bioconvertible, OptEase (Cordis), Gunther Tulip, Celect, VenaTech, Option, and Greenfield (Boston Scientific) filters are among the models that have been investigated. 

Kalva et al. examined the safety and efficacy of the TrapEase IVC filter and found that 7.5% of patients developed PE following placement, with one death attributed to PE among the 751 patient cohort. Further findings included: filter fracture in 3.0%, thrombus trapped within the filter in 25.2%, thrombus extending beyond the filter in 1.5%, and near caval occlusion in 0.7%, with no case of filter migration [62]. This study concluded that the TrapEase vena cava filters are effective at preventing PE and can be placed with minimal complications. Tsui et al. also studied TrapEase filters, reporting breakthrough PE in 1.5% of patients. Recurrent DVT (18.7%) and filter fracture (13.3%) were among the other complications observed. Although the instance of filter fracture was moderately high, the authors note that there were no cases of free fracture fragments or distant migration [63]. Other filter models demonstrating similar reduction in PE include: Denali [64], Recovery [65], Sentry bioconvertible [9,10], OptEase [66,67], Gunther Tulip [68,69], Celect [70,71], Convertible VenaTech [11], and Option [72]. Greenfield filters were examined for safety and efficacy by Kazmers et al. in a single-center study. This study reported a major complication rate of 1.3%, with mean survival time following placement of 4.96 years (n=151), concluding that Greenfield filters could be placed safely with a low rate of misplacement [73]. Recurrence of PE, complications, and general findings of these studies are outlined in Table 1. 

**Table 1:** Comparison of filter types in terms of PE recurrence and complications PE: Pulmonary Embolism, IVC: Inferior Vena Cava, DVT: Deep Vein Thrombosis, CT: Computerized Tomography

Filter Type	PE Recurrence	Complications	Conclusion
TrapEase Kalva et al., [62] Tsui et al., [63]	7.5% n=751	Filter fracture: 3.0% Trapped thrombus: 25.2%	TrapEase effective at preventing PE with minimal complications.
	1.5% n=582	Recurrent DVT in 18.7% of patients. Filter fracture in 13.3%.	PE breakthrough rates were similar to other filters despite the double basket design.
Denali Stavropoulus et al., [64]	3% n=200	No incidences of filter fracture or migration	Denali filter found to have high placement and retrieval success with few complications.
Recovery (Bard) Kalva et al., [65]	3% at mean follow-up of 63 days n=96	Penetration of IVC by filter arms in 11.5%, with fracture occurring in 3.1% of cases. No filter migration of caval occlusion.	This version of the Recovery filter has structural weaknesses leading to a high incidence of IVC wall penetration and asymmetric deployment of filter legs.
Sentry bio convertible Dake et al., [9]	0 % at 1 year 2.4% at 2 years n=129	No filter related complications	Sentry filters are effective at preventing PE, with a high rate of intended bioconversion and few complications
OptEase Ziegler et al., [67] Kalva et al., [66]	0% at 6 months n=150	Filter migration: 0.9% Symptomatic DVT: 0.8%	OptEase filters provide protection from PE with a stable amount of complications between 1 and 6 month follow-ups.
	No PE on CT at 20 month follow-up n=71	Symptoms of PE developed in 15% of patients after filter insertion. No cases of filter migration, caval wall occlusion, or filter tilting.	OptEase filters can be successfully used to prevent recurrence of PE with an acceptable complication rate.
Gunther Tulip Given et al., [68] Looby et al. [69]	0.3% PE n=317	3 filter placements resulted in minor transient abnormalities	Gunther Tulip filters can provide significant protection against PE with limited complications if removed within the appropriate retrieval window.
	0.7% PE n=147	Pneumothorax occurred in 2.7% of patients and filter expansion occurred in 0.7% of patients.	Gunther Tulip retrievable filters can be used safely and with minimal complications.
Celect Sangwaiya et al., [70]	2.8% at mean follow-up of 68 days n=73	Significant filter-tilt in 6.5% of placements Filter-related problems in 39% of cases.	Celect filters can be placed safely and reduce incidence of PE, but show a high risk of caval filter leg penetration.
Celect Platinum Lee, Brian et al., [71]	2.6%-7.7% of PE (one confirmed and two additional on follow-up CT pulmonary angiogram) n=335	IVC perforation in 19.4% of cases. New in-filter thrombus in 8.7% (8.1% nonocclusive, 0.6% occlusive). Filter tilt in 1.2%. Filter migration in 0.3%. No instances of filter fracture.	Complication rates of the Celect Platinum filter are in line with those of other models.
VenaTech Lin et al., [11]	0% at 6 months n=149	IVC perforation in 1.3% of converted No clinically significant filter migration, filter fracture, or IVC thrombosis in converted patients 14.3% IVC thrombosis in non-converted cases.	At 6 month follow-up, the converted version of the VenaTech filter showed low risk of PE and minimal adverse effects. Non-converted configuration showed a higher rate of IVC thrombosis.
Option Johnson et al., [72]	8% within 180 days n=100	2% filter migration 3% symptomatic caval thrombosis	Option IVC filters can be placed safely while maintaining a high rate of clinical success.
Greenfield Kazmers et al., [73]	Not used as endpoint n=151	1.3 % major complication rate 0.7% filter misplacement	Greenfield filters can be placed safely with minimal risk of misplacement or complication. 30-day mortality rate of 6.6% with a mean survival time of 4.96 years following insertion.
Greenfield vs. TrapEase Usoh et al., [74]	Not a primary endpoint n=84 (Greenfield) n=72 (TrapEase)	Thrombosis of either the iliac or IVC occurred in 7% of the TrapEase cohort. No incidence of filter migration, access-site thrombosis, or IVC perforation in either group.	TrapEase filters showed a higher risk of thrombosis than the Greenfield IVC filters.
Gunther Tulip (GT) vs. Trap/OptEase (TE/OE) vs. ALN vs. VenaTech (VT) Filters Koizumi et al., [76]	Embolization into the pulmonary arteries occurred in one of the two cases of filter fracture in the GT group, and three out of 19 cases in the TE/OE group. ALN and VT groups had no incidences of fracture or PE.	Filter fracture occurred in 0/19 in the ALN group, 0/2 in the VT group, 2/270 (0.7%) in the GT group, and 19/135 (14.1%) in the TE/OE group.	TE/OE filters have a high frequency of complication and are not well suited for long-term or permanent insertion.
Celect vs. Gunther Tulip vs. Greenfield McLoney et al. , [75]	Not an endpoint n=255 (Celect) n=160 (GT) n=50 (Greenfield)	Perforation was seen in 49%, 43%, and 2% in Celect, GT, and Greenfield filters, respectively. Filter fracture occurred in 0.8%, 0.6%, and 0% in the Celect, GT, and Greenfield groups.	Greenfield filters had a significantly lower rate of perforation than Celect and GT filters. All three models had low incidences of fracture.

Usoh et al. performed a randomized trial comparing Greenfield and TrapEase IVC filters, reporting 7% of cases with symptomatic IVC/IV thrombosis in the TrapEase cohort, and none in the Greenfield group (P=0.019). There were no instances of filter migration or perforation in either group. This study concluded that higher incidences of inferior vena cava thrombosis (IVCT) were likely attributable to the TrapEase filter and its structural characteristics promoting unfavorable hemodynamics [74]. Although the complications are characterized differently due to the comparison with Greenfield filters, the complication rates are largely in line with Kalva et al. in their research of the efficacy and safety of TrapEase filters [62]. McLoney et al. compared Greenfield, Gunther Tulip (GT), and Celect filters, noting higher perforation rates in GT and Celect (43% and 49%) than Greenfield (2%). In this study, all three models demonstrated a low frequency of filter fracture: 0.6% (GT), 0.8% (Celect), and no fractures in the Greenfield model [75]. A similar comparison was made by Koizumi et al. between GT, Trap/OptEase (TE/OE), ALN (Bormes-les-Mimosas, France), and VenaTech (VT) filters. This study found filter fracture in two cases with GT (0.7%), with one resulting in embolization to the pulmonary arteries. Filter fracture occurred with TE/OE filters in 14.1% of patients, with embolization in 2.2%. There were no incidences of filter fracture in the ALN (n=19) or VT (n=2) cohorts [76]. Kichang et al. compared retrievability and complication rates between Celect and Denali Infrarenal IVC filters at two-month indwelling. This randomized control trial (n=136) found a significantly higher rate of filter tilt >15° and strut penetration in the Celect filters (eight instances of filter tilt, 14 cases of strut penetration) versus the Denali (one instance of filter tilt, one of strut penetration) variety (P=0.033 and P=0.001, respectively). Three instances of breakthrough PE occurred in the Celect group with one occurrence in the Denali group [77]. Aggregate data seems to suggest that Greenfield filters exhibit a lower complication rate, with few instances of perforation, migration, and fracture. However, the variance in findings between these studies emphasizes difficulty in determining definitive guidelines and practices. There is agreement upon the reduction in the risk of PE, particularly in the patients with the most clear indications via the SIR guidelines [5], but complication rates vary between filters and studies. 

Kai et al. compared outcomes for patients treated with permanent IVC filter versus temporary, and found recurrence of PE in none of the 25 cases using the temporary filter, but 18% in the 17 patients in whom a permanent filter was placed (P=0.10). Mortality rate was 35% in the permanent filter group and 16% in the retrievable group (P=0.14) [78]. Although this study is limited by its size, n=42, it does corroborate many of the aforementioned studies in terms of PE risk reduction. Likewise, Van Ha et al. retrospectively compared retrievable IVC filters (GT, Recovery) with permanent filters, reporting an average implantation time of 226 days for the retrievable models and 288 days for the permanent filters. Incidence of PE was similar in both cohorts, 1.4% in the retrievable and 1% in the permanent. The authors note an increasing rate of filter placement due to the possibility of retrieval and expanding indications, but conclude that in both permanent and retrievable models the risk of complications and recurrent PE are acceptably low [79]. 

Given the spectrum of filters available, our literature review indicates there is a role for IVC filters in thrombotic treatments, particularly in individuals at highest risk for recurrent PE who may be contraindicated to anticoagulants. The caveat seems to be the window of placement and removal. Many of the removable filter types showed a significant reduction in recurrent PE, however, as noted by Given et al., once beyond the ideal clinical window, filter complications increase and retrieval becomes more difficult [68]. 

Cancer-Related 

Cancer patients are at a particularly high risk for developing thrombosis and subsequent embolism. In fact, venous thromboembolism is the second leading cause of death in cancer patients. Inherently, the risk rate for thrombus formation depends on the various types of cancer. 

In favor of IVC filters in cancer patients, Balabhadra et al. performed a population-based cohort study on 88,585 patients, of which 33,740 received an IVC filter. 5.1% of these patients developed PE after the initial DVT diagnosis. Patients with an IVC filter who did not develop PE had significant improvement in survival than those without the filter. Filter placement reduced development of PE in patients from low-risk to high-risk malignant neoplasms and did not show increase of new DVT [80]. 

Babu et al. performed a retrospective study involving a cohort of female patients with gynecological cancer. Filters were placed prophylactically for surgery and the postoperative period. Following the patients for six months, there was no evidence of PE or cases of mortality, and the filters were retrieved uneventfully [81]. Dewdney et al. performed a retrospective study on a similar cohort of gynecologic cancer patients. One hundred three out of 128 patients were identified as appropriate for analysis. Median survival time was 7.8 months after filter placement. Indications for placement included contraindication to anticoagulation secondary to hemorrhage (44%), preoperative conditions (30%) and failed anticoagulation (15%). The study found that there was no significant difference in survival time based on indication (P=0.18). However, patients that were eligible for anticoagulation therapy following filter placement did show better survival chances (HR 0.45, 95% CI 0.45-0.27, P=0.003) [82]. 

Abtahian et al. constructed a retrospective study that compared complications of IVC filters in cancer patients vs non-cancer patients. 17.7% of patients with active cancer had filter-related complications. 19.8% of noncancer patients had similar complications. However, retrieval rates were lower among the cancer cohort (28.0% vs 42.0%, P<0.001). Ultimately, this study concluded that while IVC filters are less likely to be retrieved in cancer patients, there is no greater risk for filter-related complications [83]. Since IVC filters tend to be unretrieved in cancer patients, Takase et al. examined the long-term effects of non-retrieved filters in cancer vs non-cancer patients. The study identified 150 cancer patients with non-retrieved filters and 305 in the non-cancer group. Patients with active cancer were associated with increased risk for DVT (HR 2.47, 95% CI 1.24-4.91, P=0.010) and there was no association with decreased risk for PE (HR 0.82, 95% CI 0.34-1.96, P=0.650). Comparatively, the non-cancer group was associated with decreased risk for PE (HR 0.29, 95% CI 0.09-0.93, P=0.037) and no increased risk for DVT (HR 1.73, 95% CI 0.89-3.38, P=0.108) [84]. 

Shaikh et al. compared the safety and outcomes of retrievable and permanent filters in patients with active cancer. The retrospective study followed 207 patients with permanent filters and 179 with retrievable, the majority having been placed due to contraindication to anticoagulant therapy. Recurrent VTE occurred in 20% of patients with retrievable filters and 24% of patients with permanent filters. Median survival time from filter placement was 8.9 months in the retrievable group and 3.2 months in the permanent group. Permanent filters were also found to be more costly. This study concluded that the benefit of IVC filters in cancer patients is unclear as complications do arise and there is a short survival time after filter placement [85]. 

Brunson et al. argue that IVC filters do not have a short-term survival benefit in patients with active cancer. The retrospective study highlighted 2747 active cancer patients who received a filter between 2005 and 2009. Of this cohort, 577 displayed indications for filter placement. Results yielded that there was no reduction in 30-day mortality (HR = 1.12, 95% CI: 0.99-1.26, P=0.08) or 180-day risk of PE (HR = 0.81, 95% CI: 0.52-1.27, P=0.36). There was an associated increase in risk of 180-day DVT [86]. However, this study is limited in that data on anticoagulation use was not provided, such that it is possible that more patients did have true indication for filter placement. Data also was not provided for cancer stage or filter retrieval status. Barginear et al. performed a retrospective study on 206 cancer patients with incidence of VTE to examine the efficacy of IVC filters compared to anticoagulation therapy. Overall survival was significantly greater in patients who received anticoagulation therapy (13 months) over the IVC filter group (two months). Combining IVC filter with anticoagulation produced a median survival of 3.25 months (P<0.0002) [87]. However, a possible limitation to this study is that the patients in the IVC filter group showed contraindication to anticoagulation and tended to exist in the more advanced stages of cancer. This study did use multivariate analysis to adjust for this difference 

To examine the survival time of advanced cancer patients who received IVC filters, Mansour et al. performed a retrospective analysis of 107 cancer patients, the majority of whom were late stage. Filter insertion was without complications; however, DVT in 10 patients, PE in three, and one filter thrombosis. Specifically, regarding the advanced-stage patients, median survival time was 1.31 months in the 59 patients with available survival data. Thirty-nine percent of these patients expired within a month and 67.8% in less than three months [88]. Although IVC filters are a relatively safe option when there is high risk for bleeding and PE, the benefit to advanced-stage cancer patients is not very apparent. Mahmood et al. studied filter complications in patients with metastatic carcinoma vs localized carcinoma. Metastatic patients tended to have more filter-related complications (25% vs 11%, P=0.03) and decreased retrieval rates (31% vs 58%, P=0.01). An additional finding showed that reinitiation of anticoagulation therapy, if indicated, could reduce filter-related complications (OR 0.3; P=0.005) [89].

Surgery-Related 

Bariatric surgery: Patients undergoing major surgery are at increased risk of developing DVT and may benefit from IVC filter placement prophylactically to prevent embolization to the lungs. Bariatric surgery patients carry a higher risk for VTE, but use of IVC filters in these cases remains controversial, as there are other risks associated with indwelling filters and difficulty of removal in obese patients [90]. Goldman et al. reported a positive correlation between BMI and rate of filter angulation, noting that patients with BMI >40 had a significantly higher likelihood of angulation than those with BMI <30 (OR 2.12; 95% CI, 1.07-4.21; P=0.03) [91]. Given the increased complication rate in this population, it’s important to ascertain whether prophylactic IVC filter insertion provides enough benefit to counteract such risks. 

Reddy et al. performed a large observational study (n=258,480; 1047 with IVC filter) comparing rates of in-hospital mortality and PE in patients receiving prophylactic IVC filter placement versus those without. In-hospital mortality or PE were significantly higher in the IVC filter cohort (1.4% vs. 0.4%; OR: 3.75; 95% CI: 1.25 to 11.30; P=0.019). Additionally, IVC filter placement was associated with higher rates of lower extremity or deep vein thrombosis compared to the non-filter group (1.8% vs. 0.3%; OR: 6.33; 95% CI: 1.87 to 21.4; P<0.01) [92]. Haskins et al. completed a large retrospective study (n=286,704; 2512 with IVC filter) which, similar to the Reddy study, provides evidence against IVC filters in bariatric surgery patients. In this study, DVT (0.7% vs. 0.2%; P<0.001) and 30-day mortality (0.44% vs. 0.07%; P<0.001) were both higher in the IVC filter cohort than in patients without a filter [93]. Unlike Reddy et al., this study did not find a significant difference in the risk of PE between groups. Finally, Kaw et al. performed a meta-analysis, reporting a higher rate of DVT (RR 2.81, 95% CI 1.33-5.97, P=0.007) with IVC filter insertion. No significant difference in risk of PE was found [94]. 

In contrast, Giorgi et al. reported that in high-risk patients undergoing bariatric surgery 2.0% (n=49) had nonfatal DVT and PE, with no incidences of complications related to placement or removal [95]. Likewise, IVC filters were placed prophylactically in high-risk bariatric patients in a prospective study (n=107) performed by Sheu and colleagues. Postoperative DVT occurred in three patients within a three-month follow-up (3%, 95% CI 1-9%), with one instance of low-risk acute PE (1%, 95% CI 0.3%). No major filter-related complications were reported in this study [90]. Schuster et al. found postoperative PE or DVT in 21% of patients (n=24) in a similar cohort [96]. Long-term outcomes in the bariatric surgery population were examined by Gargiulo, who reported DVT in 3.4% of cases (n=58) [97]. 

Given the contradictions between these studies, it would be difficult to make definitive statements about the use of IVC filters in the bariatric surgery population. It is noteworthy that the larger studies and meta-analysis seem to agree that IVC filter placement adds unnecessary risk of DVT, without any significant benefit in terms of reduction in PE. 

Orthopedic surgery: Major orthopedic surgeries can also be an indication for IVC filter placement. Ahmed et al. examined the safety and efficacy of IVC filters in the prevention of PE for high-risk patients undergoing total hip or knee arthroplasty. Retrospective analysis showed a lower incidence of PE in the high-risk group for patients who received IVC filters (0.8% to 5.5%, P=0.028). There was no significant relationship between filter placement and postoperative VTE, DVT, and PE in the low-risk group [98]. Patients with DVT undergoing major orthopedic surgery may also be at high risk of PE. Huang et al. studied the prophylactic placement of IVC filters for the prevention of PE in patients undergoing surgery for spinal, pelvic, or lower extremity fractures. Groups were divided into patients with above-knee DVT (AKDVT), popliteal vein thrombosis (PVT), and below-knee DVT (BKDVT). There were no instances of symptomatic PE upon postoperative follow-up and significant differences in the occurrence of thrombosis within the filter between the groups (11.04%, 11.70%, and 8.06%, respectively) [99]. Although IVC filter insertion showed minimal benefit in the lower-risk groups, there does appear to be a benefit in surgical patients with larger risk factors. 

Prophylactic Use and Trauma 

Traditionally, IVC filters are placed in patients with absolute contraindication to anticoagulation therapy when there is risk for acute venous thromboembolism. However, indications in the clinical setting have controversially extended beyond this to include prophylactic use in patients at high risk for VTE, particularly in the case of trauma. Patients with multiple trauma or in a postoperative state are at high risk to develop a thrombus but severity of injury indicates immediate anticoagulant therapy to be harmful at this time. 

Trauma patients are acutely susceptible to PE when anticoagulation therapy is not appropriate. Curtis et al. performed a randomized control trial on 42 trauma patients to examine the effects of retrievable prophylactic IVC filter on time left unprotected for PE and feasibility. It was found that the experimental group who received IVC filters showed reduced unprotected time (Control: 78.2 h [53.6-104]; rIVC filter: 25.5 h [9.8-44.6], P=0.0001). One PE occurred in the experimental group and two in the control. Eight deaths occurred in the experimental group and seven in the control [100]. In high-risk trauma patients unable to receive pharmacologic anticoagulant prophylaxis, retrievable IVC filters may provide a limited but clinically meaningful duration of protection against PE. However, without the history of VTE, IVC filter placement in patients with severe trauma still remains controversial. Polytrauma patients were reviewed retrospectively by Berber et al., who reported a recurrent PE rate of 2.2% in the filter group and 1.6% in the no-filter group, which was not found to be statistically significant. The goal of this study was to examine the use of the EAST guidelines for filter placement and concluded that these guidelines may exaggerate the indications where IVC filter insertion is beneficial [101]. Tran et al. performed a retrospective study that excluded patients with history of VTE. Of 1451 patients identified, 282 received an IVC filter and results were compared to the control. There was no association with PE (HR=0.46; 95% CI, 0.12,1.70; P=0.24) or mortality (HR=1.02; 95% CI 0.60,1.75; P=0.93) after filter placement but there was association with DVT (HR = 2.73; 95 % CI, 1.28,5.85; P=0.01) [102]. 

Ho et al. identified 223 trauma patients who received a retrievable IVC filter. After placement, 16% of these patients experienced subsequent DVT or VTE. Significant risk factors for complications included Injury Severity Score, lower extremity fractures, and delay in initiating prophylactic pharmacological therapy after filter insertion. For filters that remained beyond 50 days, complications included filter adherence to the vessel wall (4.9%), IVC thrombus (4.0%), and filter tilt or displacement (2.2%) [103]. Elkbuli et al. also considered time-to-therapy and its relation to different trauma outcomes. The retrospective study divided patients into two groups: 0-48 hours and >48 hours based on when they received the IVC filter. The authors reported that there was no change in significant differences in DVT, PE, or in-hospital mortality (P>0.05 for all), but a shorter ICU and hospital length of stay if the filter was placed within the first 48 hours [104]. 

Rosenthal et al. (2004) sought to determine the efficacy of placing temporary IVC filters guided by bedside ultrasound in patients with multiple trauma. Ninety-four patients between July 2002 and November 2003 received the OptEase filter, placement confirmed by abdominal x-ray. Three filters were misplaced in the right iliac vein (3.2%). Of the 94 patients, 19 died but death was not related to the filter. Two groin hematomas developed related to the filter (2.1%). Thirty-one filters were removed with no complications within 25 days of placement after initiation of anticoagulant therapy. Forty-four filters remained; 41 were due to contraindication to anticoagulation and three due to thrombus caught within the filter. This study determined that temporary IVC filters can be safely placed by bedside ultrasound in critical patients until further anticoagulation therapy can be started [105]. Rosenthal et al. (2006) performed another study utilizing the Gunther-Tulip (n=49), Recovery (n=41), and OptEase (n=37) filters in patients with multiple trauma. There was no PE or other significant filter-related complications in 96.8% of patients. Three groin hematomas developed related to the filter. This study suggested that the Gunther-Tulip and Recovery filters could be placed for longer indwelling times if contraindication to anticoagulation persists. The OptEase filter, however, would require repositioning after 21 days. Ultimately, the Gunther-Tulip filter remained the easiest to retrieve with longer indwell times [106]. 

The efficacy of the Gunther-Tulip filter to prevent PE in interventional thrombolytic treatments was evaluated in a retrospective study by Yamagami et al. Fifty-five Gunther-Tulip filters were placed in 42 patients. There were no placement-related complications. One patient experienced perforation and filter migration. In-dwell time ranged from four to 37 days. Retrieval was attempted in 18 patients, with one failure. Twenty-four patients kept the filter as the DVT was resistant to alternative treatment. This study confirmed the safety and efficacy of the GT filter to prevent PE during DVT treatment. It was also found to be convenient when there is contraindication to removal [107]. 

The primary benefit of prophylactic IVC filters is to reduce risk of pulmonary embolism. A systematic review and meta-analysis by Haut et al. investigated eight controlled studies focused on the effectiveness and safety of IVC filter placement in trauma patients. With IVC filter placement, evidence showed a consistent decrease in relative risk for PE (0.20 [95% CI, 0.06-0.70]; I2=0%) and fatal PE (0.09 [0.01-0.81]; I2=0%). There was no significant change in development or mortality of DVT [108]. Another meta-analysis by Bikdeli et al. examined 11 controlled studies. This study reported a reduced risk for subsequent PE in patients with IVC filters (OR: 0.50; 95% CI: 0.33 to 0.75). PE-related mortality was not significantly decreased (OR: 0.51; 95% CI: 0.25 to 1.05). There was increased risk for DVT (OR: 1.70; 95% CI: 1.17 to 2.48) and no change in all-cause mortality (OR: 0.91; 95% CI: 0.70 to 1.19) [109]. Ahmed et al. performed a meta-analysis to compare mortality rates of patients that received IVC filters for submassive or massive PE prevention to patients without filters. Patients who received filters were found to have a lower mortality rate (6.8% vs 26.3%) and a complication rate of 0.63%. Overall symptomatic PE recurrence was 1.4% [110]. This study suggests that IVC filters may be beneficial in the prevention of submassive or massive PE. 

Prophylactic IVC filters are of interest in perioperative care where there is contraindication to anticoagulation. Tuy et al. examined IVC filter placement combined with mechanical limb compression in patients who have undergone musculoskeletal tumor surgery in the pelvic or lower extremity regions. This study reported that there were no long-term complications of filter placement identified at follow-up of at least three months, with 3% of patients with DVT and none with known PE (n=81). They determined that IVC filter and mechanical limb compression is a reasonable thromboembolic prophylaxis for this patient population [111]. In a retrospective study, Avgerinos et al. compared the results of patients undergoing thrombolysis for acute iliofemoral DVT that were treated with and without adjunctive IVC filter. There was no statistical difference in complication rate or PE occurrence between groups. Risk factors associated with increased rate of embolization were female gender (OR, 5.833; 95% CI, 1.038-32.797; P=0.032) and perioperative PE (OR, 5.6; 95% CI, 1.043-30.081; P=0.054) [112]. During thrombolysis in patients with multiple risk factors, such as female gender or preoperative PE, IVC filters should be used with caution. 

Stein et al. (2020) reported that all-cause mortality among patients with acute PE was reduced from 46% to 24% with the addition of the IVC filter. Intravenous therapy in combination with IVC filter placement was found to have an all-cause mortality rate of 12%, while intravenous therapy alone showed a mortality of 42%. Both therapies were found to have a lower mortality rate than anticoagulants alone among this cohort [113]. However, another study performed by Stein et al. (2017) examined the efficacy of IVC filters in prevention of PE in patients who have suffered pelvic or long bone fractures. In a total cohort of 1,479,039, 17,661 patients received an IVC filter. All-cause mortality in the filter cohort was 2.9% while all-cause mortality in the patients who did not receive a filter was 1.1%. This study concluded that IVC filter placement did not reduce mortality in patients with pelvic or long bone fractures, and may not be useful for prophylaxis in this case [114]. Conversely, prophylactic IVC filter placement with severe pelvic and/or lower extremity fractures, intracranial injuries, and spinal cord injuries was found by Lee et al. to positively correlate with in-hospital mortality risk (OR: 0.46, P<0.01). However, this study reported a negative correlation of IVC filter insertion with both PE (OR: 5.25, P<0.01) and DVT (OR: 5.55, P<0.01) [115]. These competing results highlight the necessity for further research, and also show why it has been difficult to ascertain specific guidelines and consensus among clinicians. 

IVC filters have also been considered in prophylaxis for interventional procedures. In a retrospective study, Lee et al. examined the efficacy of a retrievable IVC filter prior to catheter-directed thrombectomy of lower extremity DVTs to prevent embolic shedding. In 22 out of 70 patients who received retrievable IVC filters, the thrombus was dislodged and caught by the IVC filter. There was no pulmonary embolism in patients with retrievable filters. The authors concluded that prophylactic retrievable IVC filters are beneficial in preventing PE during interventional treatments [116]. Tapson et al. reported on a novel device that combined IVC filter with a central venous catheter with the aim to reduce risk of PE in critically ill patients. The device was placed safely at bedside without fluoroscopy. There was incidence of proximal DVT within the first seven days but no catheter-related infection. The primary goal of prevention of PE was achieved in 100% of the patients [117]. 

Another population at risk for DVT and subsequent PE is women during pregnancy. Between 1998 and 2004, Kawamata et al. examined 11 patients with DVT or who had developed DVT before pregnancy received an IVC filter. Anticoagulation therapy was started with filter placement but discontinued during intrapartum. There were no complications during filter insertion. There was no incidence of pulmonary embolism during the pregnancy period or after delivery, and all filters were removed, with one being replaced with a permanent filter [118]. For pregnant patients at risk for DVT and subsequent PE, IVC filters may be an effective intervention to reduce incidence of PE. 

In recent years, rates of IVC filter placements have increased; however, follow-up and retrieval have not risen at a commensurate rate. Swami et al. performed a retrospective study to examine the indications and complications of IVC filters. Of the 254 cases examined, 65 were placed for absolute indication, 28 for relative, and 161 for prophylaxis. Complications appeared in 15 of the 96 cases with follow-up imaging. Only 19 filters were successfully retrieved [119]. This study suggested that prophylactic filters are being placed without strict follow-up for retrieval, which increases risk of filter-related complications such as filter migration or fracture. 

As seen in these studies, filter retrieval is contraindicated when there is massive clot formation or complete occlusion of the IVC filter. Pan et al. examined retrieval of filters containing clots in trauma patients between January 2008 and December 2015. Of 764 patients, 236 cases were positive for filter thrombus: 121 (15.8%) patients were found to have small clots, 97 patients presented with massive clots (12.7%), and complete occlusion was seen in 18 patients (2.4%). Utilizing CDT, 213 of the 236 filters were successfully retrieved without incidence of PE [120]. 

Long-Term Indwelling and Complications 

IVC perforation and indwelling complications have been shown to increase over time. Wang et al. examined long-term complications in patients with an indwelling time of at least four years. This study found significantly higher IVC perforation and fracture rates in permanent filters compared to replaceable types. The authors concluded that IVC filter complications are relatively common with longer indwelling time. Moreover, it was reported that higher rates of fracture were found with CordEase and TrapEase filters, but IVC perforation rates were higher with retrievable conical-type devices [25]. Desai et al. retrospectively examined complication rates of long-term indwelling between retrievable and permanent IVC filters. Despite the fact that patients with retrievable indwelling filters were younger (mean age 62 vs 74, P<0.0001), this group had a significantly higher complication rate than those with permanent filters (9% vs 3%), P=<0.001) at mean 20-month follow-up. Complications of the retrievable and permanent IVC filters included thrombotic (4.4% and 2.2%, P=NS) and device-related (3% vs 0.5%; P<0.006) events. Even within matched groups following propensity score analysis revealed significantly higher complication rates in the retrievable filter group (9.1% vs 3.5%; P=.0035), suggesting that long-term indwelling of these models may be ill-advised [121]. In concordance with these results, a recent retrospective study by Rauba et al. found a decreased rate of subsequent DVT (8.1% vs 11.9%; P=0.05; hazard ratio, 0.65; 95% CI, 0.42-1.00) and mortality (8.8% vs 28.8%; P<0.001; hazard ratio, 0.5; 95% Cl, 0.35-0.7) at mean follow-up time of 36±16 months for patients in whom the IVC filter was retrieved. Moreover, this study reported a negative association between longer indwelling time and likelihood of retrieval [122]. Of note, at mean follow-up 2.1 (0.68-4.78, n=112) years Ribas et al. found no cases of filter embolization, migration or fracture, but 12.5% had thrombotic complications including: filter thrombosis requiring long-term anticoagulation (4.5%), DVT (4.5%), and IVC thrombotic occlusion (3.6%). These included 57 patients with temporary filters and 55 with permanent filters [123]. Chow et al. confined their single-center observational study to include patients in whom permanent filters were placed, concluding that the permanent filters were effective at preventing recurrent PE but post-filter VTE and post-thrombotic syndrome were common, leading to a high morbidity rate. However, the authors did not attribute the morbidity to filter-related causes [124]. Iwamoto et al. found no incidences of recurrent PE, filter fracture, filter thrombus occlusion, or migration in 61 patients with permanent IVC filters and DVT during long-term follow-up (median: 18 months; mean: 28 months). There were several instances of PE and patient mortality within the first month, which was attributed to differences in underlying diseases and intracardiac thrombi [125]. Permanent IVC filters appear more appropriate for long-term indwelling and the authors suggest that, in combination with anticoagulation therapy, they can reduce the risk for fatal PE. In contrast, Lee (Jung-Kyu) et al. performed a retrospective observational study using CT imaging to determine major causes of IVC penetration and predictive factors for retrievable IVC filters. 87.6% incidence of IVC perforation (n=45) was reported, with associated longer in-dwelling time and diminished IVC diameter. Moreover, patients with indwelling time of >20 days were reported to have a 15.8 times greater risk of IVC penetration [39]. It would seem important to ascertain if the frequency of perforation would persist over a larger patient population, but each aforementioned study is in agreement about the dangers of lengthy in-dwelling times. Jaberi et al. sought out patients with retrievable filters that had still not been removed at median 3846.9 days since placement, concluding that, given the frequency of complications of long-term indwelling, filter removal should be performed if possible within the regulatory guidelines. This study concluded that IVC filter retrieval could be performed safely with low morbidity and mortality, reporting a success rate of 93% [43]. It is interesting to note that there seems to be general agreement regarding the dangers of long-term indwelling, however, when referencing regulatory guidelines there remains a need for consensus of best practice both in terms of indications and removal. Our review suggests that, even in longer indwelling periods, retrievable IVC filters can be removed safely. 

IVC filter abutment against the IVC wall is one of the most common reasons for filter failure. Causes of abutment were investigated by Lee et al. who reported via multiple logistic regression model that a filter-tilt angle above 9.25° and external compression are independent risk factors (ORs: 4.56, 10.18, respectively). The authors conclude that CT imaging before IVC filter placement may help reduce the risk for filter tilt and external compression leading to fewer complications associated with abutment against the IVC wall [26]. Adding to this discussion, Laidlaw et al. found that larger filter diameter was associated with higher risk for filter tilting (P=0.0004). Moreover, greater filter tilt and prolonged dwelling time were more likely to require advanced retrieval techniques (P=0.01 and 0.002, respectively) [126]. Filter fracture frequency is also associated with longer indwelling times. Vijay et al. found median dwelling time for filter fractures of 692 days (range, 61-1,771 days), with no fractures found in filters in place for less than 61 days [127]. A prospective study performed by Ho et al. studied filter changes from prolonged indwelling. Filters from 100 patients were examined following removal at a median of 54 days in situ, reporting a positive correlation between duration in situ and loss of metallic elasticity of filter struts (Pearson correlation coefficient, 0.232; P=0.008) [128]. This change leads to a higher risk of filter fracture and reinforces the need for prompt removal. 

Falatko et al. focused their study on patients >60 years old with IVC filters, and compared groups based on those who received anticoagulation therapy against those without. Mortality was the primary endpoint examined with 0.4 deaths per 1000 filter days and 0.7 deaths per 1000 filter days in the anticoagulated versus non-anticoagulated groups, respectively (P=0.06). The study concludes that the effect of anticoagulation was not significant in this cohort and notes age as a major confounding factor. Although anticoagulation is the treatment of choice for prevention of acute PE, in the elderly population there may be diminishing benefits [129]. 

Bistervels et al. studied women with indwelling IVC filters who become pregnant, reporting a complication rate of 5%. Complications were evaluated within six weeks postpartum and included filter migration, fracture, penetration, or filter thrombosis. Due to the low complication rate, the authors suggest that it can be safe to become pregnant with an indwelling IVC filter provided the filter is intact without signs of perforation. However, due to the few cases (n=20) the authors caution against making firm conclusions about the safety of IVC filters in pregnancy [130]. IVC filters can also be placed during pregnancy in patients with thrombotic risks. Konishi et al. retrospectively studied pregnant patients in Japan in whom a temporary IVC filter was placed due to risk of DVT. Among this patient group, there were two cases of filter complications with one related to an allergy to lidocaine while the other was a dislocation to the right atrium. Patients with IVC filter insertion experienced a later onset of DVT (22 vs 12 weeks; P=0.002) requiring a shorter duration of unfractionated heparin (16 vs 28 weeks; P<0.001), with no cases of PE occurring during the perinatal period [131]. The low reported complication rates suggest IVC filters may have utility in pregnant women who may have contraindications to anticoagulation or those in an acute window of risk for thromboembolic event. 

Physiology can also play a role in filter complications. Laborda et al. examined IVC filters during Valsava maneuvers, reporting a 60% decrease in IVC cross-sectional area with an accompanying five-fold increase in pressure (P<0.001). Physical changes led to increased risks of filter strut fracture, abutment, and penetration [132]. These hemodynamic and mechanical pressures are more likely to occur if the IVC filter remains in situ for a prolonged period of time, thus increasing a patient’s risk for filter-related complications. Although long-term indwelling exposes patients to further complications, these physiological variables could also be used to reduce the strain on the IVC filter. Hemodynamic changes and the inherent risks of patients requiring an IVC filter leaves them susceptible to infection. Prolonged indwelling increases this risk. Chua et al. compared the likelihood of bloodstream infection in patients with VTE in whom an IVC filter had been inserted versus patients without. Although the authors hypothesized that IVC filter implantation would coincide with higher risk for bloodstream infection, multivariable regression modeling showed no increase within one year post-VTE diagnosis in patients with IVC filters (10.7% vs 8.8%; P=0.12) [133]. 

Medicare claims data from years 2012-2016 shows a significant increase in the percentage of filter removal, but one must wonder why retrieval has not become the predominant outcome if the filters can be removed safely and the detriments of lengthy indwelling are apparent [12]. Non-retrieval of IVC filters has been suggested to be in part due to poor follow-up with patients [37]. Juluru et al. addressed the increasing numbers of retrievable, yet long-term indwelling, filters by proposing a filter-tracking software to reduce administrative burden while facilitating management of patients following IVC filter placement. The software would generate and track expected re-assessment dates, with options to update removal status, extension temporarily or permanently, and track survival status of patients. The software was tested at a single institution, and over the course of six months, tracked placement of 293 IVC filters and 83 retrievals. Compared to a control period of six months in the previous year, they found that with software facilitation, total retrieval rate was 34% compared to a control retrieval rate of 23%, though there was no significant difference in the retrieval time <210 days from placement (88.9 days control vs 102.7 days test; P=0.32). Of the filters retrieved >210 days after placement, filters were removed in the control group at a mean of 368.8 days, vs 242.5 days (P=0.03) [44]. A similar study was performed by Mikhael et al. which also found improved adherence to practice guidelines, noting an increase in retrieval rate (148/297 [49.8%] vs 223/715 [31.2%], P=0.0001) and a reduction in indwelling complication rates (30/319 [9.4%] vs 115/715 [16.1%], P=0.005) when using an automated reminder system [134]. This suggests that implementing a longitudinal tracking method may increase the overall number of filter retrievals. Importantly, improved tracking and follow-up of patients with indwelling IVC filters could potentially provide insight on any potential associated long-term consequences for these patients. 

Implications 

Results from this systematic review suggest that there is a place for IVC filters in the reduction of recurrent PE for high-risk patients contraindicated to anticoagulation. There may also be a mortality benefit in these situations as well. This seems to be the most common clinical use with the broadest acceptance in practice. A portion of the studies indicate a possible role for IVC filters for certain surgical patients. There does not appear to be broad agreement on the benefits in these cases and further studies are needed. Moreover, even in the cases when IVC filters are indicated and beneficial, it is important to note that the longer they remain indwelling, the more complications begin to accumulate. A majority of research concurs that there is positive correlation between indwelling time and complication rate, so it’s important to keep in mind the relatively high rate of retrievable filters that are not retrieved [12,25,26,37,39,43,44,121-134]. Finally, as has been mentioned in many studies regarding IVC filters, there remains a need for randomized control trials to determine their efficacy, particularly in regard to some of the expanding indications included in this review. 

Articles reviewed in this paper are summarized and listed numerically and alphabetically (Table 2). Additional information included: year of publication, country of study origin, design and study population, study duration where available, major findings, and conclusions. 

**Table 2:** Preferred Reporting Items for Systematic Reviews and Meta-Analyses (PRISMA) table showing articles on Inferior Vena Cava Filter AND complications IVC: Inferior Vena Cava, IVCF: Inferior Vena Cava Filter, PE: Pulmonary Embolism, VTE: Venous Thromboembolism, DVT: Deep Vein Thrombosis, CT: Computerized Tomography, PTS: Post-Thrombotic Syndrome, PTE: Pulmonary Thromboembolism, ROC Analysis: Receiver-Operating Characteristic Analysis, VTCF: VenaTech Convertible Filter, CDT: Catheter-directed Thrombolysis, PEVI: Percutaneous Endovenous Intervention, CVC: Central Venous Catheter, IVCT: Inferior Vena Cava Thrombosis

#	Reference	Country	Design and Study Population	Findings	Conclusions	Sub-category
	Abtahian, Farhad et al., (2014) [83]	USA	Retrospective Review (n=666) Duration: 2 years	In cancer patients, indwelling IVCF related complications occurred in 17.7% of cases versus 19.8% in the patients without cancer (P=0.50). Patients with cancer were also less likely to have their filter retrieved (28.0% vs 42.0%, P < .001).	IVCF placement in cancer patients is not associated with a higher risk of complications, but is associated with lower rate of retrieval.	Cancer Related
	Ahmed, Osman et al, (2020) [98]	USA	Retrospective Review (n=2857)	In the high-risk group of patients undergoing hip or knee arthroplasty, IVC filter was found to reduce the incidence of PE (0.8% to 5.5%, P = 0.028).	IVC filters are associated with lower incidence of PE in high-risk patients undergoing hip or knee arthroplasty.	Surgery related
	Ahmed, Osman et al, (2020) [110]	USA	Systematic review and meta-analysis (n=232)	Patients who received IVCF for massive or sub-massive PE were found to have a lower rate of mortality (6.8% vs. 26.3%) and a complication rate of 0.63% percent.	IVCF may be useful for PE prevention, but further prospectively designed studies are necessary.	Prophylactic use/Trauma
	Akhtar et al., (2018) [60]	USA	Retrospective study (n=7119)	National Inpatient Sample database identified patients with or without IVCF placement in adjunct to catheter-directed thrombolysis for proximal lower extremity or caval DVT. No significant difference in in-hospital mortality. Patients with IVCF placement had a significantly higher rate of hematoma (3.4% vs 2.1%, P=0.009) but higher in-hospital costs (P<0.001) and duration of admission.	In patients treated for lower extremity or caval DVT by catheter directed thrombolysis, adjunctive IVC filter placement does not significantly improve mortality outcomes while increasing patient burden of increased hospital stay and associated costs.	Treatments and Outcomes
	Avgerinos, Efthymios et al, (2014) [112]	USA	Retrospective chart review (n=80)	Patients undergoing thrombolysis for acute iliofemoral DVT were treated with and without adjunctive IVCF and the results were compared. No PE was found in either group, and no statistical difference in complication rate was found between groups.	IVCF should be used selectively during thrombolysis in patients with multiple risk factors, including patients with preoperative PE, women, or multiple risk factors for DVT.	Prophylactic Use/Trauma
	Babu, Suresh et al., (2013) [81]	UK	Retrospective cohort study (n=38) Duration: 2 years	In patients with gynecological cancer undergoing surgery, prophylactic IVCF placement resulted in no clinical complications, incidences of PE, or mortality at 6-month follow-up.	The authors conclude that IVCF placement is a safe method for prophylaxis in the prevention of PE for patients with gynecological cancer on chemotherapy who are undergoing surgery.	Cancer related
	Baheti, Aparna et al, (2019) [24]	USA	Retrospective chart review (n=51)	Analysis of Suprarenal IVCF placement and complications showing no incidence of indwelling filter fracture. Also, no significant change in craniocaudal position, lateral tilt, or renal function between placement and retrieval (p < 0.05)	Suprarenal IVCF placement is associated with a low complication rate and can be used safely.	Methods of Filter Placement
	Balabhadra, Samyuktha et al., (2020) [80]	USA	Population-based cohort (n=88585) Duration: 9 years	In cancer patients with acute lower extremity DVT, 5.1% of patients in whom IVCF was placed experienced subsequent PE, with improvement in PE-free survival in the IVCF versus the non-filter group (hazard ratio, 0.69; 95% CI, 0.64-0.75; P < .001).	For cancer patients with DVT and bleeding bleeding risk factors, IVCF placement can improve PE-free survival rate.	Cancer Related
	Barginear, Myra F et al., (2009) [87]	USA	Retrospective cohort study, (n=206) Duration: 2 years	Cancer patients at risk for VTE were treated with anticoagulants (AC) only (n=68), IVC filter only (n=97), and a combination (n=36). Median overall survival was 13 months with AC only, 2 months with IVC filter only, and 3.25 months with both (P<0.0002).	Anticoagulant therapy is associated with better survival outcomes than patients with contraindications requiring IVC filter. Randomized trials are needed to determine whether the poor outcomes of patients receiving IVC filters are due to a worse prognosis.	Cancer Related
	Berber, Onur et al, (2017) [101]	UK	Retrospective study (n=1138) Duration: 6 years	The IVCF placement rate was below the indication proposed by the EAST guidelines of practice. Retrievable IVCF were placed in 4.6% of cases, while EAST guidelines suggested filter insertion in 24.6% of cases (kappa concordance value of 0.103).	The authors concluded that the EAST guidelines may be overestimating the need for IVCF insertion, referencing the difference between PE with and without IVCF insertion of 2.2% to 1.8%, respectively.	Prophylaxis and Trauma
	Bikdeli, Behnood et al., (2017) [109]	USA	Systematic review & meta-analysis (n=4,204)	11 studies met inclusion criteria (6 RCTs and 5 prospective studies). Patients with IVC filters were at lower risk for PE (OR: 0.50), increased risk for DVT (OR:1.70), lower PE mortality (OR:51), and no difference in mortality (OR:91).	There are few prospective studies to assess the safety and efficacy of IVC filters. The filters seem to lower the risk for subsequent PE, increase risk for DVT, and have no significant effect on mortality.	Prophylactic/Trauma
	Bistervels, Ingrid M. (2021) [130]	Amsterdam	Retrospective Cohort (n=7, additional 13 from literature review) Duration: 20 years	Retrospectively evaluated the complications of becoming pregnant with IVCF in situ, noting a complication rate of 5%.	Becoming pregnant with IVCF in place appears to be a low risk provided the filter is intact without signs of perforation. Imaging studies should be performed to confirm the filter is asymptomatic. However, the authors note that there are a limited number of cases and broad conclusions should be avoided.	Long-term Indwelling and Complications
	Brunson, Ann et al., (2017) [86]	USA	Retrospective population-based study (n=14000) Duration: 4 years	In patients with cancer hospitalized for VTE,, IVFC provided no reduction in 30-day mortality (HR = 1.12, 95% CI: 0.99–1.26, p = 0.08) or 180-day risk of subsequent PE (HR = 0.81, 95% CI: 0.52–1.27, p = 0.36).. Filter use showed an increased risk of subsequent DVT in these patients (HR = 2.10, 95% CI: 1.53–2.89, p b 0.0001).	IVCF provided no benefit regarding short-term mortality or subsequent PE prevention. Moreover, IVCF placement increased risk of recurrent DVT.	Cancer Related
	Choi, Sun-ju et al., (2018) [21]	USA	Retrospective observational study (n=78)	Femoral vs transjugular venous approaches for placement of Denali IVC filters were compared using post-placement and pre-retrieval CT imaging. Mean fluoroscopy duration was greater in the jugular approach vs right/left femoral access (64 +/- 21s vs 67 +/-15s, p<0.05. No significant difference in measured filter tilt and filter tip abutment.	Denali IVC filters can be safely placed by either transjugular or right or left femoral access approaches.	Methods of Filter Placement
	Chow, et al., (2015) [124]	Hong Kong	Retrospective study (n=109)	Permanent IVC filters placed in patients with previous history of VTE (DVT, PE, or IVC thrombosis). Variable duration of follow-up averaging 36 months. 29.3% developed new or recurrent VTE within 2 months of filter placement. 44.6% experienced Post-Thrombotic Syndrome (PTS). Any anticoagulation therapy improved survival rate (P=0.0002)	IVC filter placement may lower risk of PE in patients with documented history of VTE. However, further studies are needed to assess risk that long term complications may arise with permanent filters.	Long-term indwelling and complications
	Chua, Abigail et al., (2020) [133]	USA	Retrospective cohort (n=4053 without IVCF, n=635 with IVCF) Duration: 3 years	There was no difference in the risk for subsequent bloodstream infection following diagnosis of VTE between the IVCF group and the non-filter group (10.7% vs 8.8%; P = .12).	In patients with newly diagnosed VTE, there is no association between the IVCF placement and risk of subsequent bloodstream infection.	Long-term Indwelling and Complications
	Clements, Warren et al., (2022) [41]	Australia	Retrospective cohort (n=124) Duration: 3 years	In a comparison of IVCF with and without complications, neither group experienced IVC thrombosis. Breakthrough PE occurred in 1.6% of cases without anticoagulation and 3.5% with anticoagulation (1 case in each group).	Use of prophylactic anticoagulation during IVCF indwelling should not be strictly guided by the presence of a filter, but by the presence of a confluence of thrombotic risks.	Treatment and Outcome
	Curtis, et al., (2020) [100]	Canada	Randomized controlled trial (n=42)	High risk trauma patients who had retrievable IVCFs placed demonstrated a clinically meaningful reduction (>24 hrs) in time vulnerable to development of PE (p=0.0001).	In high risk trauma patients unable to receive pharmacologic anticoagulant prophylaxis, retrievable IVC filters may provide a limited but clinically meaningful duration of protection against PE.	Prophylactic/Trauma
	Dake et al., (2019) [9]	USA	Prospective study (n=129)	Evaluation of the short term prophylactic utility of bio convertible Sentry IVC filter in patients with or without history of DVT and/or PE. Within the 2-year course of the study, 2.4% developed new PE, 1.6% developed IVC thrombosis. 17 new or worsening DVTs were deemed to be non-filter related.	The Sentry IVC filter may lower risk of PE for high risk patients but this is limited to a 60 day period prior to designed filter bioconversion.	Comparison of Filter Types
	Desai, et al., (2014) [121]	USA	Retrospective Study (n=1234)	This study sought to compare long term complications associated with indwelling permanent vs retrievable IVC filters. Thrombotic complications were most frequent in both permanent and retrievable (4.4% & 2.2%, P=NS). Patients with retrievable filters were younger (mean age 62 vs 74, P<0.0001), had an overall higher complication rate (9% vs 3%), P=<0.001) and significantly higher number of device related complications (3% vs 0.5%, P=0.0035).	Retrievable IVC filters tend to be placed in a younger population and have a higher rate of long-term complications than permanent filters. Care should be taken to avoid long term placement of retrievable filters.	Long-term indwelling and Complications
	Dewdney, et al., (2011) [82]	USA	Retrospective study (n=103)	Indications for IVC filter placement and survival rates were analyzed In patients with confirmed gynecologic cancer. Indications were: contraindicated to anticoagulation due to hemorrhage (44%), perioperative indication (30%) and following attempted then failed anticoagulation (14%). Survival rates were not significantly different based on indication (P=0.18). Improved survival was associated with anticoagulation treatment following IVCF placement (HR 0.45, 95% CI 0.45-0.27, P=0.003).	In patients with gynecologic cancer, IVC filter placement does not significantly improve survival. Patients who are able to receive anticoagulation after IVCF placement may have marginally improved survival than those unable to receive anticoagulation but may be impacted by lower prognosis for contraindications to anticoagulation due to worsening or complicated disease.	Cancer related
	Duffett et al., (2014) [4]	Canada	Retrospective study (n=338)	Filter related complications in 20% of the population studied. One or more thrombotic complications occurred in 11% even after initiating anticoagulation following filter placement; filter thrombosis (7%), new or progressed PE or DVT (3% and 5%, respectively), or insertion complication due to thrombosis (1%). Mechanical complications also reported; incomplete deployment (1%), IVC wall penetration/injury (1%), filter fracture (0.6%), migration (0.3%).	Because of high thrombosis associated complications, further studies are needed to evaluate safety and effectiveness of IVC filters, as well as the appropriate timing of anticoagulation therapy following IVC filter placement.	Treatment and Outcome
	Elkbuli, Adel et al., (2020) [104]	USA	Retrospective review (n=513) Duration: 6 years	This study examined whether the timing of IVCF placement affected outcomes, reporting no change in significant differences in DVT, PE, or in-hospital mortality(P > .05 for all), but a shorter ICU and hospital length of stay if the filter was placed within the first 48 hours.	IVCF placement within the first 48 hours is associated with shorter ICU and hospital length of stays.	Prophylactic/Trauma
	Falatko et al., (2016) [129]	USA	Retrospective cohort study (n=152)	In a population of patients who had IVC filters placed at age 60+ for recurrent PE, there is no significant difference in incidence of mortality when comparing those who received anticoagulation therapy post filter placement vs who had not due to contraindication of anticoagulation (P=0.46). HR for age was 1.03 (CI1.00-1.06; p-value <0.0001) and HR for BMI was 0.92 (CI 0.89-0.97; p-value 0.002).	In patients with advanced age, the benefit of anticoagulation therapy after IVC placement may be reduced than for a younger population. Advanced age may provide a prognostic factor and BMI may provide a protective factor.	Long term indwelling and complications
	Ganguli, Suvranu et al., (2016) [17]	USA	Retrospective observational study (n=688) Duration: 3 years	Bedside placement generally occurred on younger patients who less often had malignancy (P < 0.001) and more commonly received prophylactic filters (P < 0.001). Placement related complications occurred in 4.3% of bedside placements and 0.6% of fluoroscopy placements. Indwelling related complications occurred more equally: DVT: P = 0.92 PE: P = 0.61 Filter Thrombosis: P = 0.82 Timing for complications was also similar (74 vs 120 days P = 0.29)	Bedside placement guided by ultrasound can be deemed safe but is associated with more placement complications compared to fluoroscopy. Both placement methods result in similar long term complications.	Methods of Filter Placement
	Gargiulo III, Nicholas J. et al., (2010) [97]	USA	Retrospective study (n=58) Duration: 8 years	In patients undergoing gastric bypass surgery in whom IVCF placement occurred, 3.4% (2/58) developed DVT.	The authors conclude that IVCF placement is a relatively benign, safe intervention which can be largely beneficial in the prevention of DVT in patients undergoing gastric bypass. Filter placement can be particularly beneficial in the obese population.	Surgery Related
	Giorgi, Marcoandrea et al., (2018) [95]	USA	Retrospective chart review (n=49) Duration: 7 years	In patients undergoing bariatric surgery, one patient in whom IVCF was placed developed DVT and PE. 98% of filters were removed without complication.	Retrievable IVCF can be placed safely and effectively as prophylaxis against venous thromboembolism in patients undergoing bariatric surgery.	Surgery Related
	Given et al., (2008) [68]	Australia	Retrospective review (n=317) Duration: 4 years	322 GT filters were placed in 317 patients. Retrieval was attempted in 205 patients, with 15 failures giving 92% success rate. Average indwelling time was 76.95 days. 19 filters were placed permanently. 3 minor complications occurred with insertion, 5 complications with retrieval.	Insertion of Gunther Tulip filters is a safe procedure. Reported retrieval times have extended beyond the recommended 14 days which may negatively affect successful retrieval rates.	Filter Types
	Goldman, Daryl T. et al., (2019) [91]	USA	Retrospective study (n=7258) Duration: 4 years	Inclusive of all BMI indices, IVCF complication rate was 2.6%. Increased BMI was associated with increased rate of filter angulation (P = .03).	Increased BMI is associated with increased rate of filter angulation, but was not indicative of other filter-related complications.	Surgery Related
	Grullon, Jenies et al., (2022) [22]	USA	Retrospective study (n=13221) Duration: 6 years	IVCF angulation occurred more frequently in filters placed via the femoral access point compared to jugular access (0.9% vs 0.34%; OR 1.46 CI 1.02-15 2.11; p=0.04).	Transjugular placement of IVCF has a lower risk of filter angulation and access site complications compared to transfemoral insertion.	Methods of Placement
	Gul, Muhammad H. et al., (2019) [50]	USA	Retrospective observational study (n=254,465) Duration: 12 years	In patients with PE with complicating cardiac issues, mortality rate was lower in the group receiving IVCF versus those without (20.9% vs. 33%; NNT = 8.28, 95% confidence interval (CI) 7.91–8.69, E-value = 2.53).	In patients with PE and acute myocardial infarction, acute respiratory failure, shock, or requiring thrombolytic therapy, IVCF insertion was associated with lower mortality rate.	Treatment and Outcomes
	Hammond, C.J. et al., (2009) [38]	UK	Retrospective study (n=516) Duration: 12 years	IVCF placement complication rate was 0.4% over this time period. Retrieval complication rate was 1%. 24-hour and 30 day mortality rates were 1% and 8%, respectively.	IVCF placement and retrieval are associated with a limited rate of complications, but there is often a lack of follow-up in these cases resulting in a dearth of insight into efficacy and safety.	Treatment and Outcomes
	Han, Kichang et al., (2021) [77]	Korea	Prospective RCT (n=136) Duration: 2 years	The authors compared Denali and Celect IVCF, finding that the Celect group had a higher rate of filter tile >15° and strut penetration (P = 0.033 and 0.001, respectively).	Denali filters have a lower rate of filter tilt >15° and strut penetration when compared to Celect filters.	Comparison of Filters
	Haskins et al., (2018) [93]	USA	Retrospective study (n=286,704, 2512 in whom IVCF was placed)	High VTE risk bariatric surgery patients who had IVC filters in place at the time of surgery did not have a statistically significant lower incidence of postoperative PE compared with a matched subgroup.	In patients undergoing bariatric surgery, prophylactic placement of IVC filters for patients deemed high risk may not have the intended protective effect against development of clinically significant PE.	Surgery related
	Haut, Elliott R et al., (2013) [108]	USA	Review and meta-analysis (n=1064)	Relative risk was 0.20 for PE in patients with IVC filter versus patients without (P=0.01).	Prophylactic IVC filter placement is associated with lower risk of PE and acute PE in trauma patients.	Prophylaxis/Trauma
	Ho, K.M. et al., (2014) [103]	Australia	Retrospective cohort study (n=2940) Duration: 5 years	IVCF in trauma patients was examined with 16% developing DVT or VTE following placement of the filter. Mechanical complications, including adherence to the IVC wall (4.9%), IVC thrombus (4.0%), and displaced or tilted filters (2.2%) became more prevalent with indwelling time longer than 50 days.	Longer indwelling time or delay in pharmacological prophylaxis increased the risk of IVCF related mechanical complications in patients who had experienced major trauma.	Prophylactic Use/Trauma
	Ho, Kwok M. et al., (2022) [128]	Australia	Prospective study (n=100)	There was a positive correlation between length of indwelling time and loss of metallic elasticity of the filter struts (Pearson correlation coefficient, 0.232; P =.008).	Prospective analysis suggests that metallic fatigue contributes to IVCF strut fractures. There is a correlation between the length of indwelling time and metallic fatigue.	Long-term Indwelling
	Huang, Junjie et al., (2021) [99]	China	Retrospective study (n=964) Duration: 3 years	In patients with fractures and DVT in whom IVCF was placed, there were no incidences of subsequent PE. Patients were divided into above-knee DVT, popliteal vein thrombosis, and below-knee DVT, with no significant difference in filter thrombosis between groups (11.04%, 11.70%, and 8.06%, respectively).	In patients with fractures and DVT awaiting orthopedic surgery, IVCF placement can prevent PE.	Surgery Related
	Isogai, Toshiaki et al., (2015) [55]	Japan	Retrospective study (n=13125)	The authors examined the effectiveness of IVCF as an adjuvant to antithrombotic therapy, reporting a lower in-hospital mortality versus the no-filter group (2.6% vs 4.7%, P < .001; risk ratio 0.55; 95% confidence interval [CI], 0.43- 0.71; risk difference 2.1%; 95% CI, 3.0% to 1.2%; number needed to treat, 48; 95% CI, 34-82).	IVCF use may decrease the rate of in-hospital mortality in patients with PE. The authors note a need for further prospective study.	Treatments and Outcomes
	Iwamoto, Yumiko et al., (2014) [125]	Japan	Retrospective cohort study (n=72) Duration: 9 years	For patients with DVT in whom permanent IVC filters were placed, there were no new symptomatic incidences of recurrent PE at 1-month follow-up.	Underlying thrombotic conditions and diseases seem to play a large role in determining the prognosis for patients with DVT, regardless of IVCF placement status. IVCF in combination with anticoagulation may reduce risk of death due to recurrent PE in patients with DVT.	Long-term indwelling and complications
	Jaberi et al., (2019) [43]	Canada	Retrospective study (n=36)	Authors identified patients with indwelling retrievable filters for follow-up and to review possibility of removal. CT imaging found asymptomatic complications; IVC occlusion (5.8%), filter fracture (11.7%), and penetration grades 2-3 (93.75%).	Retrievable IVC filters should be removed when no longer indicated in order to prevent risk of long-term complications associated with chronic IVC filters.	Long-term indwelling and complications
	Jha, V. M. et al., (2010) [53]	USA	Retrospective study (n=248) Duration: 2.75 years	Study’s population inclusive of patients admitted for acute PE and resultant in full anticoagulation therapy. Data analyzed include whether there is right heart strain, DVT, and IVC placement. 13.3% had IVC placement. IVC placement was significantly more likely in documented DVT or IVC (P<0.0001 DVT, P<0.001 in right heart strain). But whether or not IVC reduced mortality (hospitalized) was not statistically significant.	For patients with acute PE, IVC filter in adjunct to full anticoagulation therapy was more likely in patients with right heart strain or DVT. Further studies need to investigate whether the placement of IVF filters in hospitalized patients with acute PE and pre-existing diagnoses of right heart strain or DVT actually reduces mortality risk.	Treatment and Outcomes
	Jiang, Jianguang et al., (2016) [59]	China	Retrospective study (n=189) Duration: 9 years	In patients undergoing catheter-directed thrombolysis in whom IVCF was placed, there were no major thrombolytic or placement complications, and no incidence of symptomatic PE. 4.2% of patients were found to have IVCF thrombus during the catheter-directed thrombolysis procedure.	IVCF thrombus during catheter-directed thrombolysis is uncommon, and patients in whom this occurred did not require additional treatment.	Treatment and Outcomes
	Johnson, Matthew S et al., (2010) [72]	USA	Prospective multicenter clinical study (n=100)	Retrievable Option IVC filter placement resulted in 100% technical success (deployment of filter with suitable mechanical protection from PE) and 88% clinical success (technical success without recurrent PE or other complications).	The Retrievable Option IVC filter can be placed in patients at risk for PE with few complications and significant reduction in subsequent PE.	Filter Types
	Joseph and Lopera, (2021) [18]	USA	Retrospective study (n=9)	Bedside guided placement of IVC filters via portable digital radiography (DR), in ICU patients with high ICP and raised head of bed for prophylaxis (n=8) and acute DVT (n=1). One post-procedural complication was 23% filter tilt.	IVC filters can be placed safely at bedside in patients who are unable to lay supine due to high ICP following head trauma.	Methods of Filter Placement
	Juluru, Krishna et al., (2016) [44]	USA	Retrospective observational cohort Study (n=293) Duration: 6 months	A semi-automated filter tracking system was developed over 100 hours. Control and test groups were studied over 6 months. Filter retrieval for the control group was 23%. Retrieval in the test group was 34%. There was no significant difference between time of placement and retrieval (p = 0.32)	Semiautomated tracking systems for patients with IVC filters can help coordinate clinical approach and patient care. Effectiveness can be further augmented with applications designed to improve provider communication and management plans.	Long-Term Indwelling and Complications
	Kai, Ryuichi et al., (2005) [78]	Japan	Prospective cohort study (n=50) Duration: 3 years	Comparison of temporary versus permanent IVC filters in patients with PE and/or floating DVT. 18 patients were given permanent filters, 32 temporary. Mortality rate was 35% in the former group and 16% in the latter (P=0.14). PE recurrence was 18% in the permanent filter group, with no instances of recurrence in the temporary filter group (P=0.10).	Temporary IVC filters offer a safe and effective method to reduce risk of recurrent acute Pulmonary Thromboembolism (PTE). Moreover, implantation during the acute phase of PTE reduces the risk of thrombosis. Long-term studies considering the benefits of placement against long-term complications must still be done.	Filter Types
	Kalva, Sanjeeva P. et al., (2006) [62]	USA	Retrospective study (n=751) Duration: 4 years	Examination of the efficacy and safety of the TrapEase IVC filter in a 4-year span of a single medical center. 7.5% of patients developed symptoms of PE and 1 death was attributed to PE.	The TrapEase filter was effective at preventing pulmonary embolism. Complications and recurrent PE were found to be low.	Comparison of Filter Types
	Kalva, Sanjeeva, P. et al., (2006) [65]	USA	Retrospective study (n=96) Duration: 1 year	Recovery IVCF were placed in 96 patients, with 12 developing symptoms of PE, and only 1 of the 12 having PE on CT imaging. Filters were successfully removed in 82% of patients.	No incidences of PE were found in the patient group, but there were high occurrences of asymmetric deployment of filter legs, fractures of the filter, and asymptomatic caval penetration.	Comparison of Filter Types
	Kalva, Sanjeeva P. et al., (2010) [66]	USA	Retrospective study (n=71) Duration: 5 years	At 20-month follow-up of patients who received an OptEase IVCF, 15% had symptoms of postfilter PE and 11% had symptoms of DVT. None of the aforementioned patients had PE on CT.	OptEase filters are effective against PE and have an acceptable long-term complication rate.	Comparison of Filter Types
	Kaw, Roop et al., (2013) [94]	USA	Systematic review & meta-analysis (n=102767)	Search was narrowed to adult patients undergoing bariatric surgery with or without IVCFs to assess postoperative outcomes. Incidence of DVT and mortality was found at higher significance of patients with IVC filters (P = 0.007, P = 0.1 respectively). There was no significant difference in risk of PE.	Patients undergoing bariatric surgery with IVC filters in place are at a higher risk for postoperative DVT and death. Risk of PE with or without IVC filters could not exclude a benefit in this study. Randomized control trials are recommended before IVC placement.	Surgery Related
	Kawamata, Kazuya et al., (2005) [118]	Japan	Retrospective study (n=11) Duration: 6 years	Temporary IVC filters were placed prophylactically in pregnant women with DVTl, with the authors reporting no incidences of placement or retrieval complications, nor occurrence of subsequent pulmonary thromboembolism.	IVCF may reduce risk for pulmonary thromboembolism in pregnant patients with DVT.	Prophylaxis/Trauma
	Kazmers, Andris et al., (2002) [73]	USA	Retrospective study (n=151)	Greenfield IVC filters were placed in 151 patients (152 cases) with an overall 30-day mortality rate of 30 days. Mean survival rate after filter placement was 4.96 years.	Greenfield filters can be placed safely and reduce the incidence of recurrent PE in patients with contraindications to anticoagulant therapy.	Comparison of Filter Types
	Kim, Hyoung Ook et al., (2016) [23]	Korea	Retrospective study (n=21)	In patients with lower extremity DVT, both IVC filter placement (OptEase) through the popliteal vein and PEVI were performed during a single session. Six patients (28.6%) had rethrombosis found in follow-up. Filter tilt ≥15° found in 3 patients.	IVC filter insertion through the popliteal vein can be done safely with PEVI in a single session for patients with lower extremity DVT to reduce PE-risk due to PEVI.	Methods of Filter Placement
	King, Ryan W. et al., (2021) [37]	USA	Retrospective study (n=12,874) Duration: 6 years	The authors performed a multivariable analysis which determined the independent indicators for IVCF thrombosis as new or propagated deep vein thrombosis at follow-up ( [HR], 16.3; 95% [CI], 9.8-27.3; P<0.001), no antiplatelet therapy at follow-up (HR, 4.8; 95% CI, 1.9-12.5; P=0.001), internal jugular venous access (HR, 2.2; 95% CI, 1.4-3.5; P=0.001), the presence of VTE on admission (HR, 2.7; 95% CI, 1.4-5.1; P =0.002), and temporary IVCF placement (HR, 2.5; 95% CI, 1.1-5.6; P=0.031).	Multivariate analysis suggests that given the indicators for IVCF thrombosis, antiplatelet therapy should be considered after IVCF placement to decrease the risk of IVCF thrombosis.	Treatments and Outcomes
	Koizumi, Jun et al., (2018) [76]	Japan	Retrospective study (n=532) Duration: 12 years	IVCF fracture occurred in 0.7% of Gunther Tulip filters and 14.1% of TrapEase/OptEase (P<0.001).	TrapEase/OptEase filters have a high incidence of filter fracture and are not suitable for long-term/permanent insertion.	Comparison of Filter Types
	Kolbel, Tilo et al., (2008) [58]	Sweden	Retrospective study (n=40) Duration: 12 years	In patients undergoing catheter-directed thrombolysis in whom IVCF was placed, 45% developed visible emboli within the filter. There were no incidences of symptomatic PE or filter-related complications.	In patients undergoing catheter-directed thrombolysis, thrombus embolization is common and IVCF placement can help reduce the risk of silent and symptomatic PE.	Treatments and Outcomes
	Konishi, Hiromi et al., (2015) [131]	Japan	Retrospective study (n=45) Duration: 25 years	Temporary IVCF were placed in pregnant women with DVT, resulting in a complication rate of 10%. Onset of DVT occurred later in women with filter placement compared to those without (22 vs 12 weeks; P= .002).	Pregnant women with bleeding risks may be a subset in which temporary IVCF placement could be beneficial to prevent DVT. The authors report a need for a larger prospective study to examine this conclusion.	Surgery Related
	Laborda, Alicia et al., (2014) [132]	Spain	Prospective cohort study (n=101)	After valsalva maneuver, measured 60% increase in IVC cross sectional area and 5x increase in IVC pressure (P<0.001)	Reduction in cross sectional area is associated with higher risk of filter penetration	Long-Term Indwelling and Complications
	Laidlaw, Grace et al., (2021) [126]	USA	Retrospective cohort study (n=252) Duration: 4 years	Larger IVC diameter was associated with a greater filter tilt (p = 0.0004). Greater tilt and longer indwelling times were associated with more advanced retrieval techniques (p = 0.01 and p = 0.002 respectively)	Larger IVC diameter can be used to predict greater filter tilt change which leads to complications during retrieval.	Long term indwelling and Complications
	Lee, Brian E. et al., (2017) [71]	USA	Retrospective Review (n=562) Duration: 3 years	Celect IVCF placement resulted in one case of subsequent PE and two indeterminate cases found on follow-up CT angiography (2.6%-7.7%).	Outcomes in this study were similar to those reported for first-generation Celect filters.	Filter Types
	Lee, Jung-Kyu et al., (2014) [39]	Korea	Retrospective Observational Study (n=45) Duration: 9 years	Receiver-Operating Characteristic (ROC) curve analysis was used to predict IVC filter penetration based on indwelling time and 1/IVC diameter. AUC for indwelling time was 0.855 (P< 0.001) and had sensitivity of 80.8% and specificity of 78.9% to predict penetration. AUC for 1/IVC diameter was 0.647 (P<0.048). IVC diameter of 24.2 mm was estimated to have sensitivity of 73.1% and specificity of 68.4%	Penetration on CT was common for patients with a retrievable filter. Significant penetration was associated with indwelling times > 20 days and IVC diameter < 24.4 mm.	Treatment and Outcomes
	Lee, Sang Yub et al., (2018) [26]	Korea	Retrospective Cohort Study (n=221) Duration: 8 years	IVCF tilt angle, presence of external compression, and IVC morphology were different between filter tip abutment and non-abutment groups (P<0.05).	There was a significant difference between the filter tip abutment and non-abutment groups regarding filter tilt angle, external compression, and IVC morphology. Identifying these factors may help guide filter placement away from dangerous areas.	Long term indwelling and complications
	Lee, Scott J. et al., (2022) [115]	USA	Retrospective Study (n=11938) Duration: 7 years	Prophylactic IVCF placement in trauma patients was negatively associated with in-hospital mortality risk(OR: 0.46, P<0.01), but positively associated with PE (Odds Ratio (OR): 5.25, p < 0.01) and DVT (OR: 5.55, p < 0.01).	There was a positive correlation between prophylactic IVCF placement in trauma patients with PE and DVT, but a negative association with in-hospital mortality. The authors conclude that further research is needed to examine the benefits and detriments of IVCF prophylaxis in this patient group.	Prophylaxis and Trauma
	Lee, Seul-Hee et al., (2015) [116]	Korea	Retrospective study (n=70)	Retrievable IVC filters were implanted prior to catheter-directed thrombectomy of symptomatic lower extremity DVT with or without PE to manage procedure related embolic shedding in conjunction with oral anticoagulation and stocking therapy. 31.4% of patients had thrombus trapped by IVC filter. At the end of the study period, 75.7% remained indwelling. No patient developed new PE.	In patients with symptomatic lower extremity DVT who undergo catheter directed thrombectomy. IVC filter implantation can capture thrombus and may possibly contribute to prevention of development of PE in combination with anticoagulation and stocking usage. Further randomized studies are needed to elucidate benefits attributable to IVC filters.	Prophylactic use/Trauma
	Liang, Nathan L. et al., (2016) [52]	USA	Retrospective study (n=263,955) Duration: 3 years	IVCF placement in patients with acute PE did not significantly decrease the mortality risk versus untreated patients (HR 0.93, 95% CI[0.89-1.01]).	The authors report that in patients with acute PE, IVCF does not significantly improve mortality outlook after accounting for survivor treatment selection bias.	Treatments and Outcomes
	Lin, Lawrence et al., (2020) [11]	USA	Prospective study (n=149)	IVC thrombosis occurred in 4 out of 28 patients with non-converted VenaTech Convertible Filter (VTCF), and zero out of 76 with converted VTCF (P=0.006).	VenaTech convertible filters in the convertible configuration reduced complications and risk of thrombosis and may be useful for patients with ongoing risk of VTE.	Filter Types
	Lin, Peter H. et al., (2002) [16]	USA	Retrospective study (n=592) Duration: 13 years	IVCF complication rates were not significantly different when placed by interventional radiologists versus vascular surgeons (2.1% to 2.4%, P=.48). Similarly, there was no significant difference in mortality rate between placement by interventional radiologists compared to vascular surgeons (3.8% to 3.6%, P>1.0).	Both interventional radiologists and vascular surgeons are qualified to place IVCF with no significant differences in complication rate or mortality.	Methods of placement
	Liu, Yang et al., (2021) [54]	China	Systematic review and meta-analysis (n=1274)	IVC filter does not significantly reduce PE associated mortality within 3 months of placement or entirety of patient follow-up. IVC filter is associated with lower risk of new PE within 3 months and entirety of patient follow-up (P=0.001).	IVC filter placement may reduce new occurrences of PE without significantly reducing risk of mortality, new DVT, or major bleeding.	Treatment and Outcomes
	Looby, S. et al., (2006) [69]	Ireland	Retrospective study (n=147) Duration: 4 years	Gunther Tulip IVCF were placed in 147 patients with no occurrence of subsequent IVC thrombotic events. There were 4 instances of pneumothorax, 1 instance of failure of filter expansion, and 1 incident of breakthrough PE.	The authors conclude that Gunther Tulip retrievable filters can be used safely, with a mean retrieval time of 33.6 days and minimal complications.	Comparison of Filter Types
	Mahmood, Syed et al., (2016) [89]	USA	Retrospective cohort study (n=154) Duration: 2 years	Patients with metastatic carcinoma had an increased rate of filter complications versus patients with localized cancer (25% vs 11%, P = .03). Lower filter-related complication rates were also noted in patients able to accept anticoagulation concordantly with IVCF (odds ratio, 0.3; P = .005).	Patients with metastatic carcinoma are at increased risk of filter-related complications, and whenever possible, anticoagulation should be re-initiated to reduce the likelihood of these complications.	Cancer Related
	Mansour, Asem et al., (2014) [88]	Jordan	Retrospective analysis (n=107) Duration: 7 years	Patients with stage IV cancer in whom IVCF was inserted had a mean survival time of 1.3 months, with 67.8% of patients living less than 3 months.	IVCF may be used in cancer patients if anticoagulation is not an option, however, providers should consider the stage and life expectancy of the patient and the complication/benefit ratio the filter may provide.	Cancer Related
	McLoney, Eric D. et al., (2013)	USA	Retrospective review (n=465) Duration: 1987 days	IVCF perforation was more likely in women, (45.5%) compared with men (30.8%; P = .002), and those with pre-existing malignancy compared to those without (43.7% to 29.9%; P = .001).	In comparison of Celect, Tulip, and Greenfield IVC filters, there was no significant difference in perforation rate between Celect and Tulip, with Greenfield filters having the lowest rate of perforation.	Filter Types
	Mellado, Mertixell et al., (2016) [56]	Spain	Retrospective cohort study (n=59,663) Duration: 14 years	For patients with VTE recurrence presented as DVT, there was no significant difference in death for filter insertion vs no filter (17.7% vs 12.2%, p = 0.56). For patients with VTE recurrence that presented as PE, there was a significant decrease in all-cause death for filter insertion vs no filter (2.% vs 25.3%, p = 0.08)	IVC filter insertion was not associated with survival benefit for patients with recurrent DVT in the first 3 months of anticoagulant therapy. Filters were associated with a lower risk in all-cause death for patients with recurrent PE.	Treatment and Outcomes
	Mikhael, Bassem et al., (2019) [134]	USA	Prospective study (n=1070) Duration: 2 years	The authors tracked retrieval and complication rates using a computer reminder program in patients with who had an IVCF placed previously, noting increased retrieval rate (49.8% vs. 31.2%, p = 0.0001), and reduced indwelling complication rate (9.4% vs. 16.1%, p = 0.005), in the “reminder provided” group versus the “reminder not provided group”.	This study concludes that adding an email reminder system leads to a higher rate of IVCF retrieval and a lower indwelling complication rate.	Long-Term Indwelling and Complications
	Miyahara, Takuya et al., (2006) [40]	Japan	Retrospective study (n=33) Duration: 5 years	Retrievable IVCF were placed in 33 patients with mean∓ SD indwelling time of 10.6∓ 7 days. There were no cases of PE during filter protection or retraction, but major filter-related complications occurred in 27.3% of patients.	The retrievable IVCF were effective at preventing PE, however, many complications occurred during the protection period.	Treatments and Outcomes
	Muriel, Alfonso et al., (2014) [57]	Spain	Prospective cohort (n=40142) Duration: 30 days	In patients with acute symptomatic venous thromboembolism and a significant bleeding risk, IVCF was shown to decrease all-cause mortality compared with no-insertion (6.6% vs. 10.2%; p = 0.12). PE-related mortality was also reduced in this group (1.7% vs. 4.9%; p = 0.03).	In patients with acute symptomatic venous thromboembolism and a significant bleeding risk, IVCF is associated with lower mortality risk versus no-filter placement. However, the limitations of the study prevent declaration of a causal relationship.	Treatment and Outcomes
	Nazzal et al., (2010) [33]	USA	Retrospective study (n=400)	Analysis of all patients who had an IVC filter placed at a single center, with a focus on complications. Early complications (during or immediately post-operatively) were minor and associated with hematoma and ecchymosis (0.4% patients) and late complications (months to years) include IVC thrombosis (4.75%), PE (1.5%), ipsilateral limb thrombosis (3.8%) and filter migration (1.5%). IVC thrombosis incidence was significantly higher specifically for TrapEase filters compared to other filter types (25% vs 0%, P<0.05) in a subset of hypercoagulable/malignant disease.	A single institution can safely place a variety of IVC filters for different indications without procedural or immediate post-operative conditions, while achieving no significantly different outcomes. However, a specific subset of patients with hypercoagulable states or malignant disease may experience a higher rate of IVC thrombosis with a TrapEase filter.	Treatments and Outcomes
	Pan et al., (2016) [120]	China	Retrospective Cohort Study (n=764) Duration: 8 years	In the initial pre-retrieval venogram, 236 patients were found to have thrombus within the filter 12-39 days after placement. Complete occlusion was seen in 18 cases. Retrieval was attempted in 121 cases of small clots, 120 retrievals were successful. Larger thrombi and complete occlusions were treated with Catheter-directed Thrombolysis (CDT). Overall, 213 cases were treated for thrombus without incidence of PE	Small clots are able to be retrieved without additional management. Larger occlusions can be pre-treated with CDT to assist in retrieval. Filter retrieval with manual negative pressure aspiration thrombectomy seemed valuable for management of larger occlusions.	Prophylaxis/Trauma
	Ramakrishnan, Ganesh et al., (2022) [42]	USA	Retrospective study (n=14784) Duration: 7 years	Immediate and delayed filter complication rates were 1.8% and 3.1%, respectively.	Immediate and delayed filter complications were not associated with increase in mortality. IVCF complications can be minimized by prompt filter removal.	Treatments and Outcomes
	Rauba, Jason et al., (2022) [122]	USA	Retrospective cohort study (n=1709) Duration: 6 years	Rates of subsequent DVT (8.1% vs 11.9%; P=.05; hazard ratio, 0.65; 95% confidence interval, 0.42-1.00) and mortality (8.8% vs 28.8%; P < .001; hazard ratio, 0.5; 95% confidence interval, 0.35-0.7) were lower in patients who had IVCF removed at mean follow-up time of 36 ±16 months. There was no significant difference in rates of PE between the groups.	This study compared rates of retrieval success, subsequent DVT, mortality, and PE in patients with and without filter retrieval, reporting that longer indwelling times and failure of filter removal resulted in higher complication rates and less retrieval success.	Long-term Indwelling
	Reddy, Satyajit et al., (2019) [92]	USA	Observational study (n=258,480) Duration: 10 years	The authors report that in patients undergoing bariatric surgery, prophylactic IVCF placement resulted in higher risk of in-hospital mortality or PE than in patients without filter (1.4% vs. 0.4%; odds ratio: 3.75; 95% confidence interval [CI]: 1.25 to 11.30; p = 0.019).	Prophylactic placement of IVCF may not be beneficial in patients undergoing bariatric surgery, as both mortality and complication rates were higher.	Surgery Related
	Ribas, Jesus et al., (2020) [123]	Spain	Retrospective cohort study (n=271) Duration: 11 years	Of 271 VTE patients, 205 received a temporary IVC filter. 37 patients died during the same hospitalization period as when the filter was inserted. 111 patients later had successful retrieval of the filter. 57 patients did not have retrieval for several reasons, primarily being lack of follow-up.	Lack of follow-up is a preventable cause of long-term temporary filters in VTE patients. Structured programs should be implemented to increase retrieval rates	Long-term indwelling and Complications
	Rosenthal, David et al., (2004) [105]	USA	Retrospective study (n=94) Duration: 16 months	Filters were placed within 48 hours of admission for 83 patients. 91 filters were placed without complications at L2-3. 3 filters were misplaced in the right iliac vein but were retrieved and replaced with an IVCF within 24 hours. Clinical success was determined with vena cavograms. 44 filters were not removed: 41 in patients with continued contraindication to anticoagulation therapy, 3 filters with trapped thrombus.	In patients with multiple trauma, bedside IVCF placement serves as a safe, effective bridge until anticoagulation therapy can be initiated.	Prophylaxis/Trauma
	Rosenthal et al., (2006) [106]	USA	Clinical Trial (n=127)	Retrievable filters were placed in multitrauma patients at high risk for PE. Prior to filter retrieval, patients re-evaluated and US of lower extremities found DVT in 4 patients. Filters were also evaluated after retrieval and three filters had >25% thromboses. Four filters were not removed d/t trapped thrombus.	Temporary IVC filters placed in patients with multiple traumas may be effective PE prophylactic treatment. Though intended for temporary placement, development of thrombus within the filter may prevent removal.	Prophylaxis/Trauma
	Sangwaiya, Minal Jatiania et al., (2009) [70]	USA	Retrospective study (n=73) Duration: 11 months	Of the 61 patients whose filters were placed guided by fluoroscopy, 4 filters showed immediate tilt. During follow up, 2 patients had CT confirmed PE. Radiography of 47 patients showed no filter migration. Abdominal CT of 18 patients showed filter-related complications (penetration, fracture, etc.) in 7. 14 filters were successfully removed.	The Celect IVC filter can be safely deployed and retrieved but is associated with high incidence of caval filter leg penetration.	Filter Types
	Schuster, Rob et al., (2007) [96]	USA	Retrospective Study (n=24) Duration: 3 years	In morbidly obese patients undergoing gastric bypass surgery, IVCF placement resulted in no deaths, with 21% of patients developing post-operative PE or DVT.	Preoperative placement of IVCF is recommended in high-risk morbidly obese patients undergoing gastric bypass surgery.	Surgery Related
	Shabib, Abdullah Bin et al., (2018) [32]	Saudi Arabia	Retrospective study (n=382) Duration: 9 years	IVC filters were placed in 382 patients of a single medical center between 2007-2016 with thrombotic complications occurring in 72 (19%) and mechanical complications occurring in 7 (1.8%) of cases.	Complications stemming from IVC filters can be significant. Patients with acute VTE and contraindication to anticoagulation is a widely accepted indication for use of IVC filter, but for cases outside of this scope, indications for placement must be more clearly defined.	Treatment and Outcomes
	Shaikh, Saba S. et al., (2020) [85]	USA	Retrospective study (n=386) Duration: 4 years	The authors examined the placement of IVCF in the cancer population, noting that median time from filter placement to death was 8.9 months in the retrievable filter group, and 3.2 months in the permanent filter group.	The study concludes that indications for filter placement in the cancer population are unclear, given the short time from filter placement until death, the cost, complication rate, and risk of recurrent VTE.	Cancer Related
	Sharifi, et al., (2012) [61]	USA	Clinical trial (n=141)	Patients undergoing percutaneous endovenous intervention (PEVI) procedure as treatment for DVT had lower rate of PE development with concomitant IVC filter placement than in patients without (P=0.048).	Patients receiving PEVI as treatment for acute DVT are at high risk of iatrogenic DVT and this risk may be lowered by IVC filter placement.	Treatment and Outcomes
	Sheu et al., (2018) [90]	USA	Prospective study (n=107)	Prophylactic IVC filters were placed in patients at high VTE-risk prior to bariatric surgery. Postoperatively, DVT occured in 3 patients (3%, 95%CI 1-9%), acute low risk PE in 1 patient (1%, 95%CI 0.3%). One required thrombolysis and the rest were managed with anticoagulation therapy. Median time of filter placement was 54 days (22-1548) and no major filter related complications.	IVC filters placed prophylactically in bariatric patients at high risk of VTE, combined with chemical prophylaxis, may be relatively low risk and help prevent development of life-threatening PE.	Surgery related
	Stavropoulos et al., (2016) [64]	USA	Clinical trial (n=200)	Retrievable Denali IVC filter placed in 200 patients for PE prophylaxis and followed for 2 years post-placement . Within that time period, 3% developed PE, 13% developed new or progression of existing DVT. Three cases of asymptomatic >3 mm penetration found post-retrieval. 5% filter related infection.	Retrievable Denali IVC filter can be used for temporary PE prophylaxis up to two years with a relatively low complication rate.	Filter Types
	Stein, Paul D. et al., (2016) [45]	USA	Retrospective cohort study (n=2,621,575) Duration: 9 years	In patients over 80 years old with stable acute PE, lower mortality was reported in those who received IVCF versus the group without filter (6.1% vs. 10.5%).	Absent a randomized control trial to verify the results, IVCF placement should be considered for elderly patients with stable acute PE.	Treatment and Outcomes
	Stein, Paul D. et al., (2017) [114]	USA	Retrospective cohort study (n=1,479,039) Duration: 9 years	In patients with fractures of the pelvis or long bones, the rate of all-cause mortality was 1.1% without IVCF placement, and 2.9% with IVCF.	IVCF placement did not reduce mortality in patients with pelvic or long bone fractures, and may not be useful for prophylaxis in this case.	Prophylactic Use/Trauma
	Stein, et al., (2018) [51]	USA	Retrospective cohort study (n=814)	IVC filters were placed in hospitalized patients with recurrent PE (within 3 months). All cause mortality with IVCF was 3% and without was 39.3% (P<0.0001). In stable patients who did not have thrombolytic therapy or pulmonary embolectomy, mortality with IVCF was 2.6% and without IVC was 42.6% (P<0.0001).	Limited data suggests that IVC filter placement in hospitalized patients with early recurrent PE have decreased all cause mortality than in patients without IVCF.	Treatment and Outcomes
	Stein, Paul D. et al., (2018) [48]	USA	Retrospective cohort study (n=479) Duration: 4 years	In unstable patients with acute PE, in-hospital all-cause mortality was lower in those who received IVCF versus those who did not (19.4% vs. 40.8%, P < .0001).	IVCF placement in unstable patients with acute PE seems to reduce risk of all-cause mortality if the filter is placed within the first 1-2 days of admission.	Treatment and Outcomes
	Stein, Paul D. et al., (2017) [46]	USA	Retrospective cohort study (n=24,495) Duration: 4 years	In patients over 60 years old with acute PE and cancer, those with IVCF had a lower risk of in-hospital all-cause mortality compared to patients without filter (7.4% vs. 11.2%, P<0.0001, relative risk 0.67).	In the subset of patients above 60 years old who have PE and cancer, IVCF may reduce the risk of all-cause in-hospital and 3-month mortality.	Treatment and Outcomes
	Stein, Paul D. et al., (2019) [47]	USA	Retrospective cohort study (n=4,066,513) Duration: 15 years	In patients with PE, all-cause mortality was reduced in unstable patients with IVCF compared to without (28.8% vs. 46.3%, P<0.0001). In the stable patient group, all-cause mortality was slightly reduced in the IVCF group versus the non-filter group (5.8% to 6.5%, P<0.0001).	IVCF placement in unstable patients reduces the risk of all-cause mortality, while the frequency of filter placement in these patients has decreased. Moreover, the majority of IVCF are placed in stable patients in whom there seems to be minimal reduction in all-cause mortality.	Treatment and Outcomes
	Stein, Paul D et al., (2020) [49]	USA	Retrospective cohort study (n=2923) Duration: 5 years	In-hospital all-cause mortality was reduced in stable patients undergoing pulmonary embolectomy in whom IVCF were placed compared to those without (4.1% vs. 27%, p <0.0001). In unstable patients undergoing pulmonary embolectomy, all-cause mortality was also reduced in the filter versus non-filter group (18% vs. 50%, p <0.0001).	Both stable and unstable patients undergoing pulmonary embolectomy had a reduced risk for all-cause mortality, provided IVCF was placed within the first 4-5 days of admission.	Treatments and Outcomes
	Stein, Paul D. et al., (2020) [113]	USA	Retrospective cohort study, (n=9265) Duration: 1 year	Patients with unstable PE. Anticoagulated patients with IVC suffered lower mortality rate than those without (P<0.0001).	Adjunctive therapies in combination with IVC filter may lower mortality risk in patients with unstable PE.	Prophylaxis/Trauma
	Swami et al., (2014) [119]	USA	Retrospective study (n=254)	Majority of IVCF placement 63.4% prophylactic placement. Of these, post-traumatic = 80.1%. Asymptomatic complications in 96 filters.	Although patients with prophylactically placed IVCFs have higher survival rates, asymptomatic complications like filter migration and IVC penetration may risk development of future problems.	Prophylaxis/Trauma
	Takase, Toru et al., (2020) [84]	Japan	Retrospective cohort study (n=3027) Duration: 7 years	Among 2626 patients, with acute symptomatic VTE, a total of 455 IVCF were placed and not retrieved. In the active cancer group, non-retrieved IVCF was associated with increased risk for DVT (p = 0.010) but not with decreased risk for PE (p - 0.650). An association for decreased risk for PE (p = 0.037) was found in the non cancer stratum. This group was not associated with increased risk for DVT (p = 0.108)	There are differences in the effects of IVC filter use on VTE patients with or without active cancer	Cancer Related
	Tapson et al., (2017) [117]	USA	Clinical trial (n=163)	Nitinol IVC filter placement used in combination with central venous catheter as PE prophylaxis in acutely ill, high-risk patients. No patient developed PE or fatal PE. 30% patients developed new or worsening DVT (7% within the first week).	Using IVC filter as a short term combination prophylactic therapy with Central Venous Catheter (CVC) may prevent significant or fatal PE in acutely ill patients.	Prophylaxis/Trauma
	Tran, et al., (2020) [102]	USA	Retrospective comparative study (n=1541)	In patients with severe trauma, prophylactic IVCF placement is associated with higher hazard of DVT (P=0.01), but is not associated with in-hospital PE (P=0.24) or all-cause mortality (P=0.93)	Prophylactic IVCF placement in patients with severe trauma and no prior history of VTE may increase DVT hazard without improving risk of in-hospital PE or mortality.	Prophylactic/Trauma
	Tsui, Brian et al., (2018) [63]	USA	Retrospective study (n=594) Duration: 88 months	In patients who received a TrapEase IVCF, 1.5% experienced breakthrough PE and recurrent DVT occurred in 18.7%. Filter fracture occurred in 13.3% of cases.	Breakthrough PE rates were similar with the TrapEase filter to other models available. Instance of filter fracture was relatively high, but there were no incidences of free fracture fragment or distant migration.	Comparison of Filter Types
	Tuy, Benjamin et al., (2008) [111]	USA	Retrospective cohort study (n=81)	17 of 81 patients who underwent surgery for pelvic or lower extremity malignancies were found to have DVT when an IVC filter was placed in coordination with mechanical compression (P=0.443)	Mechanical compression in combination with IVC filter placement may be beneficial for cancer patients undergoing surgery for pelvic or lower extremity malignancy.	Prophylactic/Trauma
	Usoh, Fred et al., (2010) [74]	USA	Prospective randomized study (n=156) Duration: 2 years	156 patients were randomized into 2 groups based on filter type. 84 to Greenfield, 72 to TrapEase. In a 12 month follow up, 5 patients of the TrapEase group developed symptomatic thrombosis. None developed in the Greenfield group (P = 0.19). There was no filter migration, misplacement, or perforation.	The TrapEase filter is associated with a higher rate of symptomatic IVC thrombosis.	Filter Types
	Van Ha, Thuong G. et al., (2007) [79]	USA	Retrospective review (n=604) Duration: 5 years	In a comparison of retrievable IVCF versus retrievable filters, the incidence of PE upon follow-up in the retrievable group was 1.4% and 1% in the permanent filter group.	The authors note filters being placed more frequently due to the prospect of retrievability, but conclude that there was a low rate of clinically significant PE or filter complications.	Filter Types
	Vijay, Kanupriya et al., (2011) [127]	USA	Retrospective review (n=63) Duration: 6 years	The fracture rate for Recovery, G2, and G2 Express IVC filters was 12%, and incidence of fracture increasing with longer dwelling times. Successful removal rate of non-fractured components was 98.4%, and 53.4% for the fractured components.	Rates of IVCF fracture increase with longer dwelling times, but even instances of fracture, it is possible to remove the filters safely and effectively.	Long-term Indwelling and Complications
	Walker, John A et al., (2021) [19]	USA?	Retrospective study (n=129)	Critically ill patients who had IVCF placement at bedside with digital radiograph were exposed to less radiation (median exposure=25 mGy) compared to patient who had IVCF placement by conventional fluoroscopy (median exposure = 256.94), P<0.0001). Duration of the digital radiography procedure was longer (14.5 +/- 2 vs 6.7+/- 6 min).	Given comparable results in technical success of IVCF filter placement, digital radiograph guided placement at bedside can reduce higher levels of radiation exposure of the conventional fluoroscopy method.	Methods of Filter Placement
	Wang, Stephan L. et al., (2016) [25]	USA	Retrospective study (n=96) Duration: 3 years	Study was limited to patients who had undergone contrast CTs at least 4 years after IVCF placement. Retrievable filters had higher incidence than permanent filters for perforation of IVC with or without involvement of retroperitoneal structures (P<0.0001). Also reported incidence of filter fracture and occlusion (both partial and full).	Filter type and brand may pose higher risk of certain complications; Higher rates of fracture were observed in Cordis OptEase and TrapEase filters.	Long-term Indwelling and Complications
	Wassef, Andrew et al., (2017) [34]	Canada	Retrospective (n=464) Duration: 4 years	IVCF placements were performed in patients with contraindication to anticoagulation in 44% of cases, while 20.7% were placed were not contraindication to anticoagulation and 30.6% were placed in patients with active cancer.	IVCF placement occurred in patients beyond the normal scope of practice, possibly introducing additional risk to patients without contraindication to anticoagulation or those with active cancer.	Treatments and Outcomes
	Weinberg, Ido et al., (2014) [35]	USA	Retrospective Review (n=688) Duration: 2 years	IVCF complications were found in 17.7% of patients, with DVT being the most common complication. The study further examined the relationship between IVCF placement and the commencement of anticoagulation therapy, noting that adequate anticoagulation was initiated in 66% of patients in <3 days following filter insertion.	The authors conclude that IVCF placement can be safe, but note a fairly high complication rate, combined with lack of retrieval, and delays between starting anticoagulation and retrieval.	Treatments and Outcomes
	Yamagami, et al., (2005) [107]	Japan	Retrospective study (n=55)	Evaluation of the Gunther Tulip retrievable IVC in patients with lower extremity DVT to prevent PE. There were no procedure associated complications. One patient experienced perforation and filter migration. Average placement time of removed filters was 4-37 days and filters were left in 24 patients due to refractory DVT.	The Gunther Tulip retrievable model can be safely placed in some patients with lower extremity DVT at risk for PE. Advantageous to have an option of retrieval if possible. Stopped reading after they mentioned case series…	Prophylaxis
	Yamashita, Yugo et al., (2016) [36]	Japan	Retrospective cohort study (n=257) Duration: 8 years	Study cohort was divided into two groups: IVC filter (n = 78) and No-IVC filter (n = 179). The non retrievable filter group was associated with worse mortality rate (P < 0.01) but showed no significant difference in incidences of DCT recurrence (P = 0.07)	IVC filters were frequently associated with VTE treatment but with low retrieval rates. Indications for filter placement were primarily DVT in intrapelvic or proximal veins, less commonly contraindication to anticoagulant therapy. The authors concluded that a prospective randomized trial is needed for better analysis of indications, efficacy, and associated risks.	Treatments and Outcomes
	Xiao, Liang et al., (2012) [20]	China	Randomized clinical trial (n=108)	Patients with DVT pre-thrombolysis had Günther IVC filters placed transfemorally with or without an introducer curving technique designed to prevent filter tilting. Filter tilt reduction was statistically significant in the test group (ACF=4.4 +/- 3.2 vs 7.1+/- 4.52 deg, P=0.001). Prior to retrieval, the rate of hook adherence to the vascular wall was also reduced in the test group (2.9% vs 24.2%, P=0.025).	Introducer curving during transfemoral placement of the Günther tulip IVCF can reduce rate and degree of filter tilting.	Methods of filter placement
	Ziegler et al., (2008) [67]	USA	Randomized multi-center prospective trial (n=150)	Evaluation of OptEase retrievable filter’s safety and effectiveness as a permanent option with follow-up review at 1 month and 6 month time points after filter placement. Within that time period, 4.3% filter fracture, 11.4% filter tilting.	The risks vs benefits of using retrievable filters as a long term or permanent option needs to be better understood.	Filter Types

Limitations****

Our review was limited by the exclusion and search criteria outlined in the methods section. These criteria were used to narrow our focus to IVC filter placement and complications as they relate to thrombosis, however, there may be relevant information in research studies that did not meet the selection criteria. There may be complications related to filter placement and retrieval that are not thoroughly discussed. Additionally, because search parameters were limited to IVC filters and thrombosis or complications, there may be indications for use not included in this review. Moreover, our search was limited to three databases (PubMed, ScienceDirect, ProQuest), and may not include relevant papers accessible through other databases. Articles were excluded because full-text articles or versions in English were not available. This could result in relevant information being missed. Finally, there have been few randomized control trials performed to evaluate IVC filter efficacy and complications, making any conclusions preliminary with a need for further research. 

Evidence Limitations 

This review contains an abundance of information pertaining to IVC filters and their potential complications. However, it is not exhaustive. There are a limited number of randomized control trials related to filter placement. We have tried to highlight trends, using mostly retrospective data, but any conclusions are limited by the variability of experimental design. Some studies used mortality as a primary endpoint, while others used complications or recurrence of PE. Complications were measured during different timeframes. Most seem to agree that indwelling time is positively correlated with complications, however, measurements and timing of follow-up differ. This review is also inclusive of several articles written by Stein et al. These articles met our criteria for inclusion, but it is possible results have been more heavily influenced by these studies than by any other group of authors. 

## Conclusions

IVC filter placement and removal remains controversial, both in terms of indications for use and the safety and efficacy of treatment. While there are no consensus guidelines for best practice, there is compelling evidence that IVC filters can provide significant protection against PE with minimal complications if the treatment window is appropriate. Complications become more frequent with longer indwelling time, and despite risks of removal, evidence suggests that the benefits of retrieval outweigh the risks. There also seems to be a role for IVC filters in prophylaxis against PE. Research confirms reductions in PE for trauma patients within a bridging window and as perioperative care in coordination with anticoagulation. However, evidence demonstrates that the benefits of IVC filters tend to diminish in cases where anticoagulation is an option. Given the acute risks of PE, the use of IVC filters can be an important resource, particularly in patients with contraindication to anticoagulants. Subsequent removal is a beneficial step in the reduction of filter-related complications that would lower patient risk of mortality and morbidity. Finally, further research into the broader scope of indications may be necessary to elucidate the boundaries of when prophylactic filter placement outweighs risks. Moreover, agreement on treatment window and best practice guidelines would help improve clinical decision-making and reduce IVC filter-related complications. 
